# Wnt/β-catenin signaling promotes neurogenesis in the diencephalospinal dopaminergic system of embryonic zebrafish

**DOI:** 10.1038/s41598-022-04833-8

**Published:** 2022-01-19

**Authors:** Markus Westphal, Paolo Panza, Edda Kastenhuber, Johanna Wehrle, Wolfgang Driever

**Affiliations:** 1 Developmental Biology, Faculty of Biology, Institute Biology 1, Albert Ludwigs University Freiburg, Hauptstrasse 1, 79104 Freiburg, Germany; 2 CIBSS and BIOSS-Centres for Biological Signalling Studies, University of Freiburg, Schänzlestrasse 18, 79104 Freiburg, Germany; 3 Present Address: Department of Developmental Genetics, Max-Planck-Institute for Heart and Lung Research, Ludwigstraße 43, 61231 Bad Nauheim, Germany; 4 Present Address: Institute of Anatomy, University of Zurich, Winterthurerstrasse 190, 8057 Zurich, Switzerland

**Keywords:** Developmental biology, Neurogenesis, Developmental neurogenesis

## Abstract

Wnt/β-catenin signaling contributes to patterning, proliferation, and differentiation throughout vertebrate neural development. Wnt/β-catenin signaling is important for mammalian midbrain dopaminergic neurogenesis, while little is known about its role in ventral forebrain dopaminergic development. Here, we focus on the A11-like, Otp-dependent diencephalospinal dopaminergic system in zebrafish. We show that Wnt ligands, receptors and extracellular antagonist genes are expressed in the vicinity of developing Otp-dependent dopaminergic neurons. Using transgenic Wnt/β-catenin-reporters, we found that Wnt/β-catenin signaling activity is absent from these dopaminergic neurons, but detected Wnt/β-catenin activity in cells adjacent to the caudal DC5/6 clusters of Otp-dependent dopaminergic neurons. Pharmacological manipulations of Wnt/β-catenin signaling activity, as well as heat-shock driven overexpression of Wnt agonists and antagonists, interfere with the development of DC5/6 dopaminergic neurons, such that Wnt/β-catenin activity positively correlates with their number. Wnt/β-catenin activity promoted dopaminergic development specifically at stages when DC5/6 dopaminergic progenitors are in a proliferative state. Our data suggest that Wnt/β-catenin signaling acts in a spatially and temporally restricted manner on proliferative dopaminergic progenitors in the hypothalamus to positively regulate the size of the dopaminergic neuron groups DC5 and DC6.

## Introduction

Dopaminergic (DA) neurons form a major neuromodulatory system in the central nervous system that regulates diverse functions such as motor coordination, mood control, reward associated behaviors, and hormonal homeostasis^1,2^. Accordingly, degeneration and dysfunction of DA neurons cause severe diseases in humans^3^. There is strong interest in understanding signaling and transcriptional mechanisms of DA neuron differentiation both in vivo and in vitro to facilitate cell replacement and regenerative therapies to compensate for DA neurons loss^4–6^. Several signaling factors are used to improve cell replacement therapies, and have been selected based on their ability to promote DA neuron differentiation in vivo^7–9^.

Wnt/β-catenin signaling regulates multiple processes during midbrain DA (mDA) neuron development in mammals^10–13^. Wnt1 signals induce the mDA progenitor domain at the intersection with Shh, repress alternative neuron fates in the ventral midbrain, promote midbrain progenitor proliferation, and allow the terminal differentiation of mDA neurons^10,12,13^. Furthermore, WNT-1 and WNT-5a concomitantly regulate the balance between proliferation and differentiation in mDA progenitor cultures^10,11^. Other Wnt ligands, including Wnt7a in mice, contribute to DA neurogenesis as well as axonogenesis^14^.

Less is known about the roles of Wnt/β-catenin signaling during development of forebrain DA neurons. In zebrafish, Wnt/β-catenin signaling might be involved in the development of specific ventral forebrain DA neurons^15–17^. These include the DC2 and DC4 groups in the ventral diencephalon (posterior tuberculum), as well as the hypothalamic DC5 and DC6 groups. We use “DC” here to denote “dopaminergic clusters” different from the original nomenclature^18^, as some of these groups are not located anatomically in the diencephalon^19^. The DC2,4,5 and 6 DA neuron groups constitute the diencephalospinal system in zebrafish, and their development is dependent on the transcription factor Orthopedia (Otp)^20–24^. The zebrafish Otp-dependent diencephalospinal system is homologous to the A11 DA groups in mammals^22,23^. Wnt/β-catenin signaling initially determines the size of the ventral diencephalic DC2 DA progenitor pool within the neural plate^17^. At late embryonic and larval stages, neurogenesis from proliferating neural stem and progenitor pools determines the number of hypothalamic DC5 and DC6 DA neurons^25^, however contributions of Wnt/β-catenin signaling to the development of these neurons remain elusive. The Wnt/β-catenin signaling effector Lef1 is required to initiate neurogenic gene programs within the posterior hypothalamus of zebrafish^16^. Furthermore, Wnt/β-catenin signaling is active within the posterior recess region of the hypothalamus and controls radial glia self-renewal, proliferation, and differentiation^15,26,27^. DA neurons of the DC5 and DC6 groups lie within the hypothalamus and arise from proliferating progenitors during an extended developmental time span. Wnt/β-catenin signaling, therefore, might be involved in progenitor pool regulation and differentiation of these neurons as well.

Here, we analyze contributions of Wnt/β-catenin signaling during development of ventral diencephalic and hypothalamic Otp-dependent, A11-type DA neurons. We show that Wnt/β-catenin pathway genes and transgenic Wnt/β-catenin activity reporters are expressed in close spatial and temporal proximity to DA neuron development. Pharmacological manipulations of Wnt/β-catenin signaling, as well as overexpression of Wnt/β-catenin pathway components interfere with DC5 and DC6 DA neuron development within the hypothalamus, specifically during stages when most DC5 and DC6 progenitors are still proliferative. Our data support a model in which Wnt/β-catenin is required for progenitor cell expansion of Otp-dependent DA neurons.

## Results

### Wnt/β-catenin pathway component expression in relation to ventral forebrain DA neuron development

**Figure 1:**
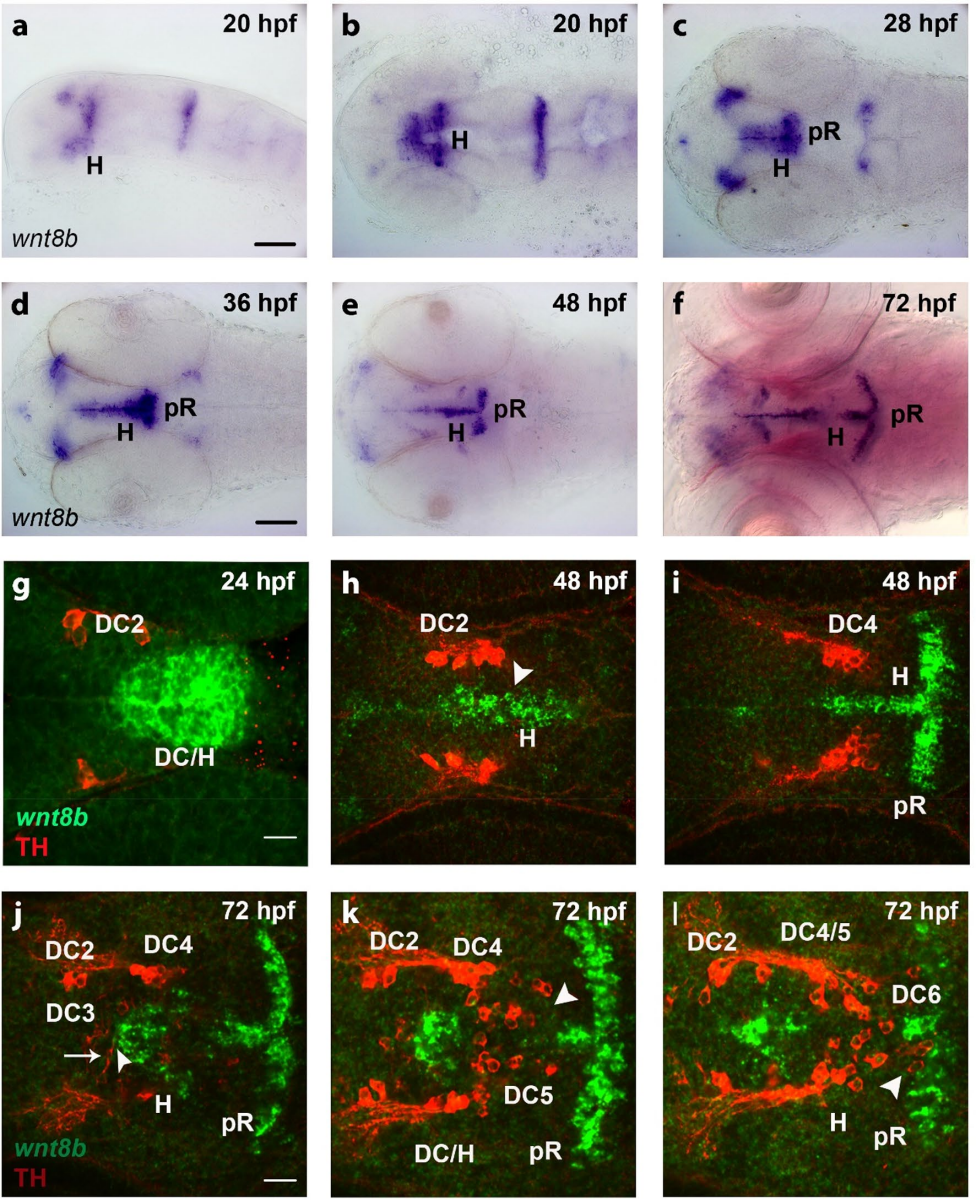
Expression of *wnt8b* in the ventral diencephalon and hypothalamus and in relation to dopaminergic neurons. (**a**–**f**) Expression of *wnt8b* in embryos as detected by whole mount in situ hybridization at stages as indicated. Lateral (**a**) and dorsal (**b**–**f**) views of heads of embryos, images generated from Z-projections of image stacks. Scale bars in (**a**) and (**d**) are 100 µm for (**a**–**f**). (**g**–**l**) Expression of *wnt8b* detected by fluorescent in situ hybridization (green) in relation to TH-immunoreactive DA neurons detected by immunofluorescence (red) in embryos at stages as indicated. Dorsal views of the ventral diencephalon/hypothalamus region. Confocal image stacks were recorded and images show single optical sections of Z-stacks containing TH-immunoreactive cells. DA neuron groups DC2 and DC4 of the ventral diencephalon and DC5 and DC6 of the hypothalamus are labeled. (**h**,**i**). Two optical sections of a single 48 hpf embryo at 13.97 µm distance from dorsal (**h**) to ventral (**i**). (**j**–**l**) Three optical section of a single 72 hpf embryo at (**j**–**k**) 13.97 µm and (**k**–**l**) 5.08 µm distances, with the dorsalmost section shown in (**j**). Scale bar in (**g**) is 20 µm for (**g**–**l**). (**g**–**l**) Z-stacks are included as video files in Supplementary Information as Supplementary Video 1 (**g**), Supplementary Video 2 (**h**–**i**), and Supplementary Video 3 (**j**–**l**). Scale bar in (**g**) is 20 µm for (**g**–**i**) and scale bar in (**j**) is 20 µm for (**j**–**l**). *DC* diencephalon, *H* hypothalamus, *pR* posterior recess.

To identify Wnt/β-catenin pathway components that might act during DA neuron development, we performed an expression analysis of Wnt ligands, receptors, and antagonists during ventral forebrain DA neuron development (Fig. 1 and Supplementary Fig. 1). We surveyed the zebrafish information network database (ZFIN; www.zfin.org) for previously described expression profiles of Wnt/β-catenin pathway genes in ventral forebrain regions where DA neurons develop (Supplementary Table 1). The Wnt ligand genes *wnt8b*, *wnt11* and *wnt16* emerged as canonical Wnt candidates (Fig. 1 and Supplementary Fig. 1b,c). Their expression in neurogenic zones and within the hypothalamus of the larval zebrafish brain have been previously reported^28^. *Wnt8b* is expressed within the diencephalon in the Zona limitans intrathalamica (ZLI) and ventral areas as well as the hypothalamus at 20 and 28 h post fertilization (hpf) (Fig. 1a,b). From 36 hpf onwards the hypothalamic expression of *wnt8b* becomes restricted to the ventricular zone and, by 72 hpf, to an anchor-like domain in the posterior recess (Fig. 1d–f). Duncan et al.^28^ described a similar expression within the hypothalamic ventricular zone and the posterior recess region for *wnt11* and *wnt16* (Supplementary Fig. 1b,c, based on^28^; *wnt11* previously named *wnt11r*). Next, we analyzed the spatial relationship between *wnt8b* expressing cells and Tyrosine hydroxylase (TH) immunoreactive DA neurons within the ventral diencephalon and hypothalamus (Fig. 1g–l). The earliest born TH-immunoreactive DC2 DA neurons arise at 20 hpf within the posterior tuberculum of the ventral diencephalon, when *wnt8b* expression is detected anterior-dorsal and posterior-ventral to these neurons, but *wnt8b* and TH do not colocalize (data not shown). At 24 hpf *wnt8b* expression is detected directly adjacent to the posteriormost TH-immunoreactive cells of DC2, now located in the forming mantle zone, in a domain extending from the midline (Fig. 1g and Supplementary Video 1). At 48 hpf, *wnt8b* expression becomes restricted to the hypothalamic ventricular zone, and the TH-immunoreactive DC2 and DC4 cells are directly adjacent to the *wnt8b* expression domain (Fig. 1h,i and Supplementary Video 2). The *wnt8b* expression domain extends rostrally along the hypothalamic ventricle and the posterior recess region, within the progenitor domain from which new TH-immunoreactive DA neuron groups in this region may develop. A similar spatial relationship is also found at 72 hpf (Fig. 1j-l and Supplementary Video 3). At 72 hpf, DC3 neurons of the medial hypothalamus are located directly adjacent to *wnt8b* expressing cells of the medial hypothalamic mantle zone (Fig. 1j, arrow points towards a DC3 neuron, and the arrowhead points towards the adjacent *wnt8b* expression domain). Interestingly, we found that some DC3 neurons extend ventricular feet towards the ventricular wall, where they may contact *wnt8b* expressing cells (Fig. 1j, arrowhead and Supplementary Video 1). Our observation suggests a direct signaling link between *wnt8b* expressing cells in the medial hypothalamus and DC3 neurons. We also observe newly formed DC5 and DC6 neurons in close proximity to the *wnt8b* expression domains of the ventricular and posterior recess regions (Fig. 1k,l and Supplementary Video 3). Some of the caudalmost TH-immunoreactive DC6 neurons appear to intermingle with *wnt8b* expressing cells of the posterior recess region (Fig. 1l, arrowhead and Supplementary Video 3). In summary, DA neurons of the ventral diencephalon and hypothalamus develop in spatial and temporal proximity to *wnt8b* expressing cells.

Our database survey also identified Wnt/β-catenin receptors and antagonists expressed in the ventral diencephalon and hypothalamus. The Wnt receptors *fzd8a* and *fzd8b* are both expressed in the forebrain in a similar pattern (Supplementary Fig. 1d–i). At 24 hpf *fzd8a* and *b* are expressed in a small stripe within the subpallial and preoptic telencephalon, and more broadly in the anterior hypothalamus (Supplementary Fig. 1d,g). The hypothalamic expression domain of *fzd8a* subsequently extends towards the posterior tuberculum and encompasses a broad region in which DA neurons develop (Supplementary Fig. 1e,f). In contrast, *fzd8b* transcripts are mostly restricted to the hypothalamus from 36 hpf onwards (Supplementary Fig. 1h,i). The extracellular Wnt antagonist *sfrp5* is expressed in the subpallial and preoptic telencephalon, the ventral diencephalon and hypothalamus at 24, 36 and 48 hpf, and becomes successively restricted to the hypothalamus (Supplementary Fig. 1j–l). Furthermore, we analyzed the expression of the Wnt antagonist *dkk1* (Supplementary Fig. 1m–o). Previous expression data from zebrafish embryogenesis stages indicate that *dkk1* expression is highly dynamic in the zebrafish neuroectoderm and is subsequently downregulated^29,30^. At 24 hpf, *dkk1* transcripts are mostly absent from neural tissue and restricted to the prechordal mesoderm (Supplementary Fig. 1m, arrowhead). We detected *dkk1* transcript in two lateral domains in the posterior tuberculum of the ventral diencephalon close to a region where DA neurons develop at 36 and 48 hpf (Supplementary Fig. 1n,o arrowheads). In summary, several Wnt/β-catenin signaling pathway components are expressed in regions where DA neurons develop in zebrafish, suggesting a role of Wnt/β-catenin signals in their development.

### Domains of active Wnt/β-catenin signaling are adjacent to ventral diencephalic and hypothalamic DA neurons

**Figure 2:**
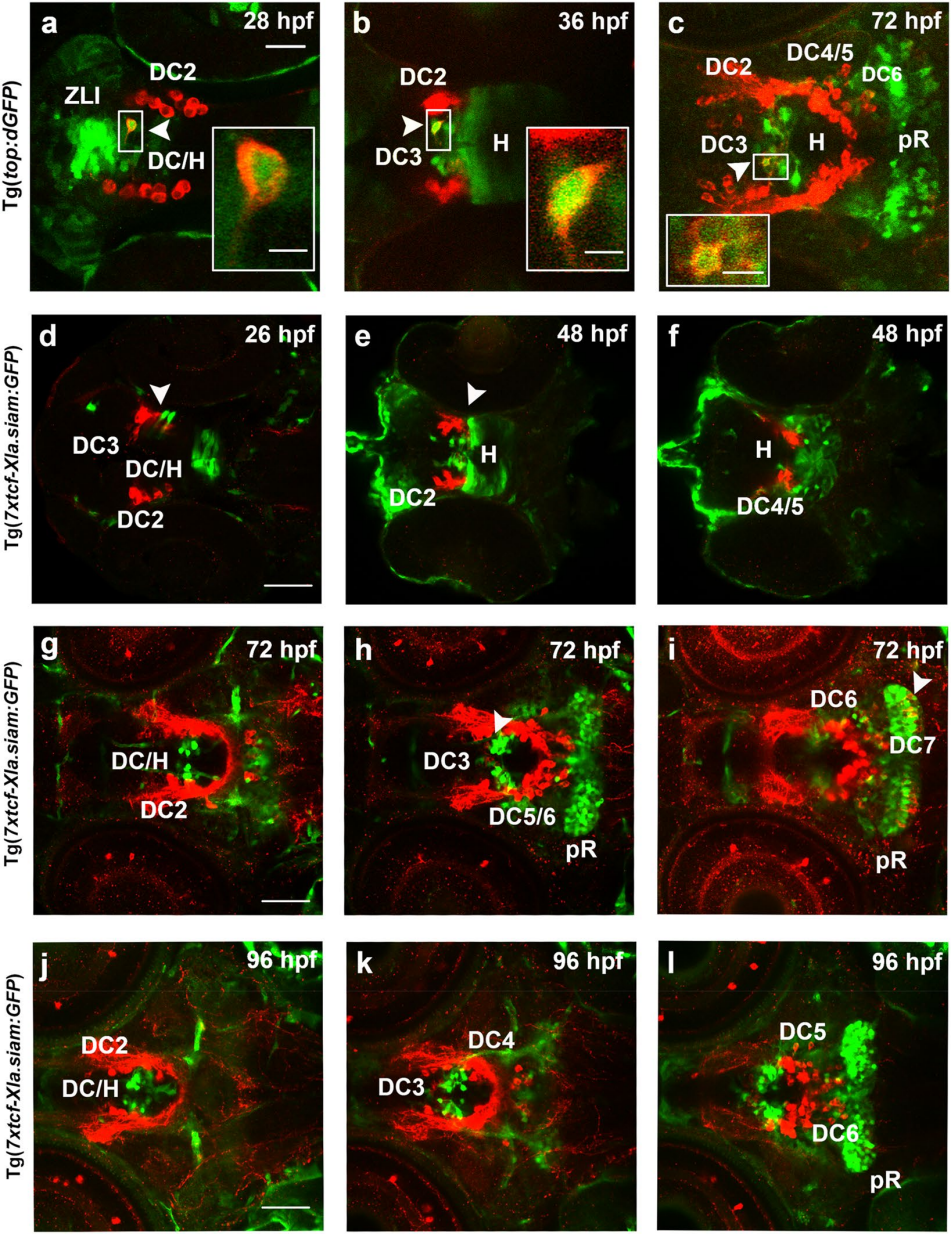
Activity domains of Wnt/β-catenin signaling in relation to TH-immunoreactive cells in the ventral diencephalon and hypothalamus. (**a**–**l**) Wnt/β-catenin-reporter Tg(*top:dGFP*) (**a**–**c**) and Tg(*7xtcf-Xla.siam:GFP*) (**d**–**l**) zebrafish embryos were stained by double immunofluorescence for TH-immunoreactive cells (red) and GFP-immunoreactive cells (green) at indicated stages. Dorsal views of the ventral diencephalon/hypothalamus region. Confocal image stacks were recorded and images show 1.2 µm single optical sections of Z-stacks containing TH-immunoreactive cells. DA neuron groups DC2 and DC4 of the ventral diencephalon, DC3 of the medial hypothalamus and DC5 and DC6 of the hypothalamus are labeled. (**e**,**f**) Two optical sections of a single 48 hpf embryos at 33.02 µm distance from dorsal (**e**) to ventral (**f**). (**g**–**i**) Three optical section of single 72 hpf embryo at (**g**–**i**) 16.51 µm and (**i**,**j**) 11.43 µm distances, with the dorsalmost section shown in (**g**). (**j**–**l**) Three optical section of a single 96 hpf embryo at (**j**–**k**) 11.43 µm and (**k**–**l**) 13.47 µm distances, with the dorsalmost section shown in (**j**). (**d**–**l**) Z-stacks are included in Supplementary Information as Supplementary Video 4 (**g**), Supplementary Video 5 (**e**,**f**), Supplementary Video 6 (**g**–**i**) and Supplementary Video 7 (**j**–**l**). Inserts in (**a**–**c**) show higher magnifications of boxed areas in (**a**–**c**). Scale bar in (**a**) is 20 µm for (**a**–**c**) and in (**d**,**g**,**j**) is 50 µm for (**d**–**l**) Scale bars in inserts are 5 µm for (**a**–**c**). *DC* diencephalon, *H* hypothalamus, *pR* posterior recess, *ZLI* Zona limitans intrathalamica.

We used the transgenic Wnt/β-catenin reporter lines Tg(*top:dGFP*) and Tg(*7xtcf-Xla.siam:eGFP*)^31,32^ to visualize domains of Wnt/β-catenin signaling activity in relation to DA neuron development. We analyzed transgenic reporter embryos by anti-GFP and anti-TH double-immunofluorescence from 24 to 96 hpf (Fig. 2). At 26 hpf, we found active Wnt/β-catenin reporter expression in neuroepithelial cells of the ventral diencephalon and hypothalamus spanning the neuroepithelium from apical to basal side (Fig. 2d; arrowhead, Supplementary Video 4). We observed this domain of Wnt/β-catenin signaling activity positioned just caudally to the early born DC2 DA neurons, which have located their somata to the basal prospective mantle region. Our data suggest that Wnt/β-catenin signaling is likely active in neuroepithelial early stem and progenitor cells within the diencephalon/hypothalamus (Fig. 2d; arrowhead). In addition, embryos of the Tg(*top:dGFP*) transgenic line show the zona limitans intrathalamica (ZLI) Wnt/β-catenin reporter activity domain to be positioned dorsal and rostral to the DC2 TH-immunoreactive DA neurons at 28 hpf (Fig. 2a). GFP expression continues in cells of the hypothalamic ventricular zone located caudally to TH-immunoreactive DA neurons at 36 hpf (Fig. 2b). At 48 and 60 hpf, the Tg(*7xtcf-Xla.siam:eGFP*) Wnt/β-catenin reporter expression expands to more posterior regions of the hypothalamus (Fig. 2e,f and data not shown). Caudal TH-immunoreactive DA neurons of the DC4 group in the posterior tuberculum are adjacent to Wnt/β-catenin reporter expressing cells (Fig. 2f, Supplementary Video 5). The TH-immunoreactive cells of DC2 are lateral to a few medially located Wnt/β-catenin reporter expressing cells (Fig. 2e). At 72 hpf and 96 hpf, expression of the Wnt/β-catenin reporter is reduced in the ventral hypothalamus (Fig. 2c,g–i,j–l), where Wnt/β-catenin responsive cells are restricted to few medially located cells. Notably, TH-immunoreactive DA neurons of the DC3 group in the medial hypothalamus intermingle with cells showing active Wnt/β-catenin dependent transcription (Fig. 2c,g,h,j,k). At 28–48 hpf, when few DC3 group cells are present, it appears that most TH-immunoreactive DC3 cells are also GFP-immunoreactive (Fig. 2a; box), while at 72–96 hpf most DC3 DA neurons are not GFP-immunoreactive, and only few express TH and GFP (Fig. 2b–c; boxes). From 72 hpf onwards, the GFP immunoreactive domain in the caudal hypothalamus expands laterally, correlating with the posterior recess region (Fig. 2c,h,i,l, Supplementary Videos 6, 7). GFP reporter expressing cells within the posterior recess region locate directly caudal to the hypothalamic DC5 and DC6 groups (Fig. 2c,h,i,k,l). At the caudal border of the DC6 group, we found some of the most posterior TH-immunoreactive DC6 to intermingle with GFP expressing cells of the posterior recess (Fig. 2c,h,i,k,l). Furthermore, we found cells of the caudal hypothalamic DC7 group located in the posterior recess to be intermingled with GFP immunoreactive cells (Fig. 2i and Supplementary Video 6). In summary, DC2, DC4, DC5 and DC6 DA neurons of the ventral diencephalon and hypothalamus arise in close proximity to cells that show Wnt/β-catenin reporter activity. In contrast some DC3 DA neurons are also GFP-immunoreactive. However, the number of DC3 GFP-immunoreactive cells diminishes at later stages, suggesting that only DC3 progenitors are exposed to Wnt/β-catenin signaling, and the GFP protein may be stable enough to be detected in early TH positive DC3 neurons, even though Wnt/β-catenin reporter transcription may be limited to progenitors. Wnt/β-catenin signaling reporter expression appears not active in terminally differentiated DC2-6 DA neurons.

### Pharmacological perturbation of Wnt/β-catenin signaling affects hypothalamic DC5 and DC6 DA neurogenesis

**Figure 3:**
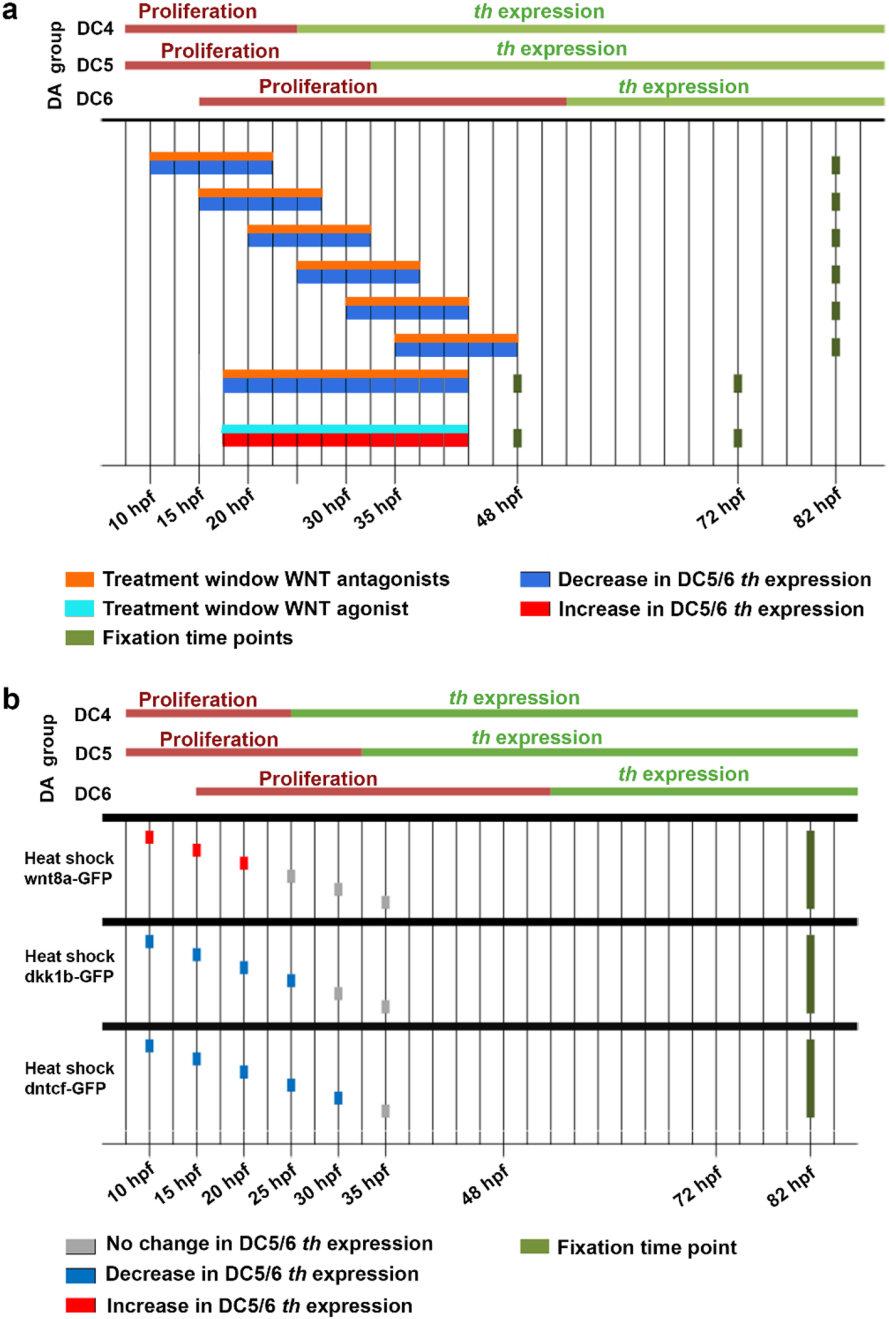
Summary of effects of inhibition and activation of Wnt/β-catenin signaling on hypothalamic DC5/6 DA neuron development. (**a**) Experimental design and summary of the results of pharmacological inhibition and activation of canonical Wnt signaling. The graph summarizes the findings from Figs. 4 and 5 and Supplementary Figs. 2 and 3. Top half of bars shows treatment time, bottom half color code for effect on DA neurons. Small molecule antagonists IWR-1 or XAV939 were applied for overlapping 12 h time windows (orange bars). The small molecule antagonist IWR-1 and the agonist BIO were also each applied from 18 to 42 hpf (orange and cyan bars). Small molecule and control treated embryos were fixed at 48 hpf for Wnt target gene *axin2* expression analysis and either at 72 hpf or 80 hpf for *th* expression analysis (dark green dots). Color code indicates whether the treatment caused a decrease (blue) or an increase (red) in *th* expression as analyzed in embryos at either 72 or 80 hpf. The upper part indicates the temporal correlation of treatment windows and results of pharmacological manipulations to cell cycle exit and differentiation of DC4 and most of DC5 and DC6 DA neurons^25^. (**b**) Experimental design and summary of the results of heat-shock induced overexpression of Wnt8a, Dkk1b and a dominant negativeTcf3 (dnTcf3). The graph summarizes the findings from Figs. 6 and 7. Single heat shock treatments were performed at indicated time points (dots). Heat shocked heterozygous transgenic embryos and WT siblings were fixed as indicated (green dots) for *th* expression analysis. Color code indicates whether the treatment caused no change (grey), decrease (blue) or an increase (red) in *th* expression. The upper part indicates the temporal correlation to DA development as in (**a**).

To determine whether development of diencephalic and hypothalamic DA neurons is affected by Wnt/β-catenin signaling, we used a pharmacological approach and perturbed Wnt/β-catenin signaling within defined developmental time windows (Fig. 3a). We used the pathway antagonists IWR-1 and XAV939 to globally inhibit Wnt/β-catenin signaling^33,34^. We applied each antagonist separately for overlapping 12-h time windows starting either at 10, 15, 20, 25, 30 or 36 hpf, and analyzed *th* expression by chromogenic whole mount in situ hybridization (WISH) at 80 hpf. To avoid a potential interference with Wnt/β-catenin signaling during early pattern formation, especially in the neural plate, we chose 10 hpf as the earliest time point to apply the antagonists. We found IWR-1 and XAV939 treatments to reduce the expression of the Wnt/β-catenin target gene *axin2* in treated embryos as compared to DMSO controls (Fig. 5a,b and Supplementary Figs. 2, 3), suggesting that inhibitor treatments reliably reduce Wnt/β-catenin signaling activity.

**Figure 4:**
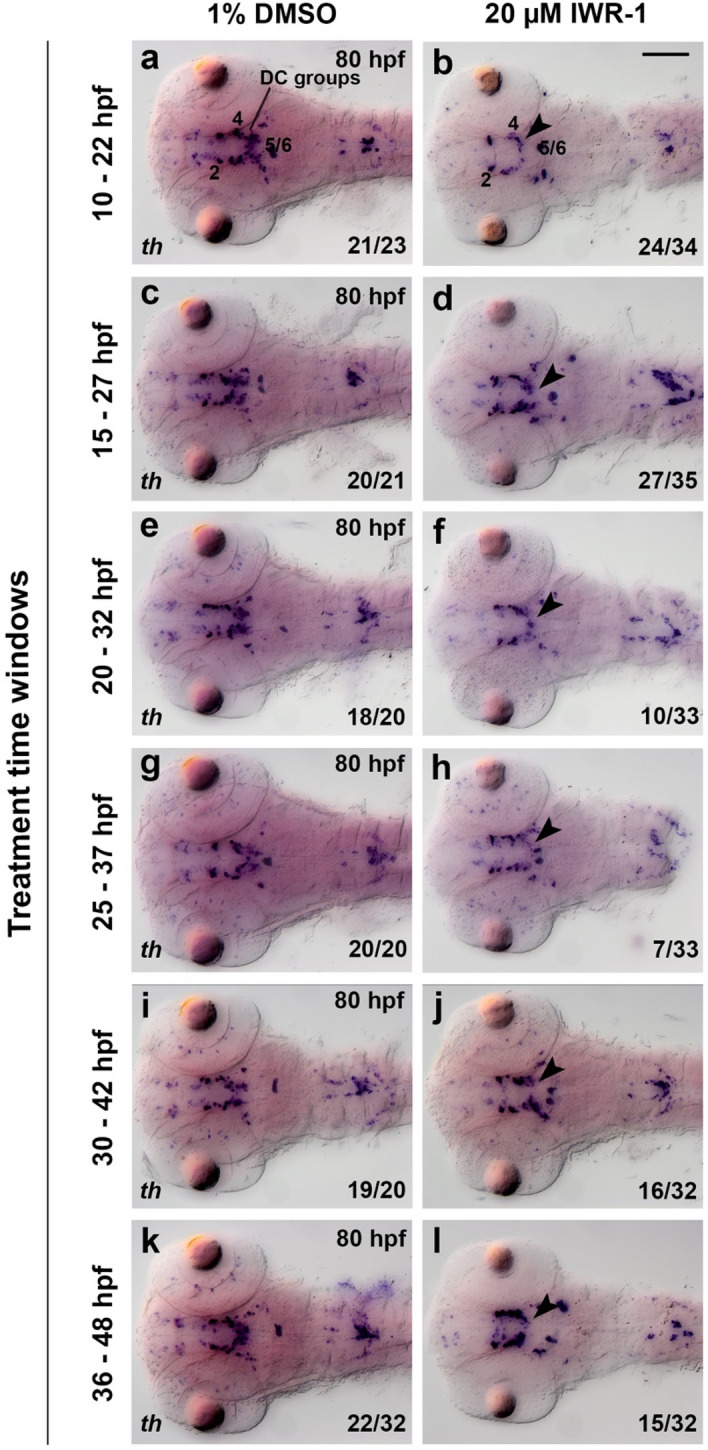
Inhibition of Wnt/β-catenin signaling by IWR-1 affects DA neurons in the ventral diencephalon and hypothalamus. (**a**–**l**) Expression analysis of the DA neuron marker *th* by whole mount in situ hybridization in embryos treated with IWR-1 (right) or DMSO (control, left) during time windows indicated on the left side of each row, and subsequently fixed at 80 hpf. The *th* expressing DC groups 2–6 are indicated in (**a**) and (**b**). The arrowhead in (**b**,**d**,**f**,**h**,**j**,**l**) point at decreased *th* expression in DC5/6 groups. Dorsal views of heads of larvae, images are Z-projections of image stacks. Scale bar in (**b**) is 100 µm for all images. Numbers N/N in bottom right corner of each image indicate number of representative phenotypes as shown in image versus total embryos analyzed for this condition.

**Figure 5:**
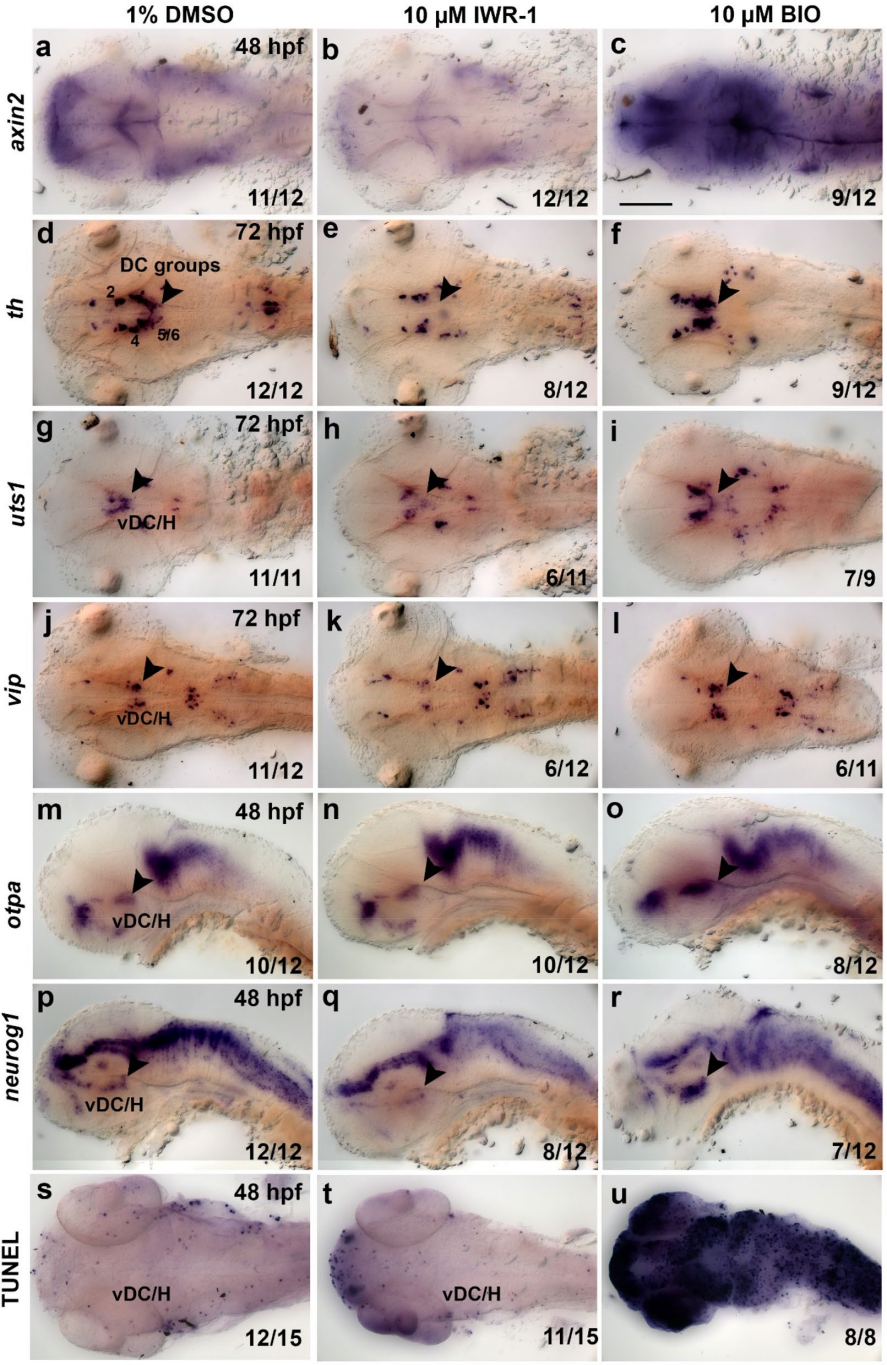
Pharmacological inhibition and activation of Wnt/β-catenin signaling affect neurogenesis of hypothalamic neurons. (**a**–**r**) Expression analysis of *axin2* (**a**–**c**), *th* (**d**–**f**), *uts1* (**g**–**i**), *vip* (**j**–**l**), *otpa* (**m**–**o**) and *neurog1* (**p**–**r**) by whole mount in situ hybridization and detection of cell death by the TUNEL assay (**s**–**u**) in embryos treated with DMSO (control), IWR-1 or BIO as indicated at top. All embryos were treated from 18 to 42 hpf and fixed at stages indicated in left column of image panels. (**a**–**l**) Dorsal and (**m**–**r**) lateral views of larval heads, images are Z-projections of image stacks. The arrowheads point to differences in marker gene expression within the ventral diencephalon and hypothalamus. Scale bar in (**c**) is 100 µm for all images. Numbers N/N in bottom right corner of each image indicate number of representative phenotypes as shown in image versus total embryos analyzed for this condition. *H* hypothalamus, *vDC* ventral diencephalon.

We focused our analysis on the DC2, DC4, DC5 and DC6 DA neurons. We did not analyze DC3 despite the interesting finding of Wnt/β-catenin reporter activity in early DC3 neurons, because DC3 DA neurons form at a slow rate during a long developmental time window^25^, which would have required long inhibitor treatments to see strong effects on DC3 neuron numbers. Extended inhibitor treatments also tend to affect development of the whole embryo, and thus potential effects on DC3 may be indirect. Further, DC3 neurons express *th* transcripts at much lower levels than the other DA groups, which makes quantifying effects on these cells difficult. We found that DC2, as well as DC4, DC5 and DC6 DA neuron groups were differentially affected by global Wnt/β-catenin inhibition (Figs. 3a, 4, Supplementary Fig. 3). Both IWR-1 and XAV939 treatments did not affect *th* WISH staining intensity in DC2 DA neurons, which become post-mitotic during neural plate stages before the earliest inhibitor treatments^25^. This finding is in agreement with a previous report showing that Wnt/β-catenin signaling restricts the DC2 progenitor pool size before 10 hpf^17^. IWR-1 treatment at 10–22 hpf reduced *th* expression in DC4, DC5 and DC6 DA groups (Fig. 4a,b, arrowhead). However, during the same treatment window, XAV939 only affected *th* expression in the DC5 and DC6 DA groups (Supplementary Fig. 3c,d, arrowhead). We observed reduced *th* expression in DC5 and DC6 after IWR-1 and XAV939 treatments at 15–27 hpf and 20–32 hpf (Fig. 4c–f; Supplementary Fig. 3g,h,k,l, arrowheads). IWR-1 treatments at 30–42 hpf and at 36–48 hpf also caused reduced *th* staining intensity in DC5 and DC6 groups (Fig. 4i–l), but did not have a significant effect on DC4 neurons in this treatment window. DC4 neurons are postmitotic and differentiated already before 30 hpf^25^. We also treated embryos with IWR-1 during a prolonged time window from 18 to 42 hpf and observed a strong reduction in Wnt/β-catenin target gene *axin2* expression at 48 hpf (Fig. 5a,b) as well as a complete loss of *th* expression in DC5 and DC6 groups in treated embryos at 72 hpf (Fig. 5d,e, arrowhead). Loss of DC5 and DC6 neurons in IWR-1 treated larvae might not be attributed to enhanced cell death, since we do not observe an increase in apoptotic cells within the ventral diencephalon and hypothalamus as analyzed by TUNEL staining (Fig. 5s,t). In summary, pharmacological inhibition of the Wnt/β-catenin signaling pathway impairs *th* expression in, or development of, DA neurons of the hypothalamic DC5 and DC6 groups during time windows when their progenitors are still proliferative. The early differentiating posterior tubercular DC4 group is affected only during the earliest treatment window, suggesting that Wnt/ß-catenin inhibition also affects progenitors for this group.

Next, we analyzed effects of pharmacological activation of the Wnt/β-catenin signaling pathway. We treated zebrafish embryos using the Glycogen Synthase Kinase-3 (GSK3) inhibitor and Wnt/β-catenin pathway agonist BIO^32,35^. We performed treatments during a broad time window (18–42 hpf), and analyzed *th* expression at 72 hpf (Fig. 3a). Expression of the Wnt/β-catenin target gene *axin2* was increased in BIO treated embryos analyzed at 48 hpf, demonstrating effective Wnt/β-catenin signaling overactivation (Fig. 5a,c). We observed a strong increase in *th* staining intensity and potentially cell number in DC5 and 6 groups in treated embryos at 72 hpf, as compared to DMSO controls (Fig. 5d,f arrowhead). This increase in DC5 and 6 groups is despite the finding that cell death, as judged by TUNEL staining, is enhanced in BIO treated embryos (Fig. 5s,u). In summary, pharmacological activation and inhibition of the Wnt/β-catenin signaling pathway lead to opposite effects on development of hypothalamic DC5 and DC6 DA neurons.

Since Wnt/β-catenin signaling has been shown to control neurogenesis broadly in the zebrafish hypothalamus^15,16,27^, we tested the effect of pharmacological modulation of Wnt/β-catenin signaling on other hypothalamic neurons. We chose *uts1* and *vip* expressing neurons, because of their proximity to DC5 and DC6 DA neurons within the hypothalamus^19,36^. IWR-1 treatments at 18–42 hpf led to a reduction of *uts1* and *vip* expression in the hypothalamus at 72 hpf (Fig. 5g,h,j,k). BIO treatments during the same time window resulted in the opposite effect, showing an increase in *uts1* and *vip* staining intensity in the hypothalamus at 72 hpf (Fig. 5g,i,j,l). Thus, neurons that reside in proximity to hypothalamic DC5/6 DA neurons are similarly affected by Wnt/β-catenin signaling modulation.

To further investigate whether pharmacological Wnt/β-catenin modulation influences DA progenitor populations within the ventral diencephalon and hypothalamus, we stained treated and control embryos for *otpa* and *neurog1* transcripts, which are expressed in progenitors and are required for DC5 and DC6 DA neuron differentiation^22,37,38^. We observed a slight decrease in both *otpa* and *neurog1* expression in embryos treated with IWR-1 from 18 to 42 hpf and analyzed at 48 hpf (Fig. 5m–q, arrowheads). In contrast, embryos treated with BIO during the same time window showed increased expression of *otpa* and *neurog1* in the ventral diencephalon and hypothalamus at 48 hpf (Fig. 5m–r, arrowheads). We therefore conclude that Wnt/β-catenin signaling controls expansion of DA progenitor populations in the ventral diencephalon and hypothalamus.

We further tested whether BIO and IWR-1 treatments displayed an early effect on Sox2 immunoreactive neural stem or progenitor cell proliferation in the hypothalamus. We treated embryos between 15 and 34 hpf with BIO or IWR-1, and labelled S-phase cells by EdU incorporation at 34 hpf. The embryos were fixed at 36 hpf and stained using an anti-Sox2 monoclonal antibody, which recognized both Sox2 and Sox3 protein in zebrafish (Supplementary Fig. 4a–c), and also stained to detect EdU incorporation (Supplementary Fig. 4d-f). We counted the number of Sox2 single and Sox2/EdU double positive nuclei and calculated their percentage of all Sox2 positive nuclei (Supplementary Fig. 4g). Following BIO treatment we find a significantly increased percentage of EdU labelled Sox2 cells, while IWR-1 resulted in no change. In summary, the BIO activation suggests that Wnt/β-catenin signals may affect proliferating neural stem and/or progenitor cells, while the IWR-1 data on *otpa* and *neurog1* would indicate that DA progenitors may also be affected.

### Overexpression of Wnt8a, Dkk1b and ΔTcf3 affect hypothalamic DC5 and DC6 DA groups in a stage-dependent manner

**Figure 6:**
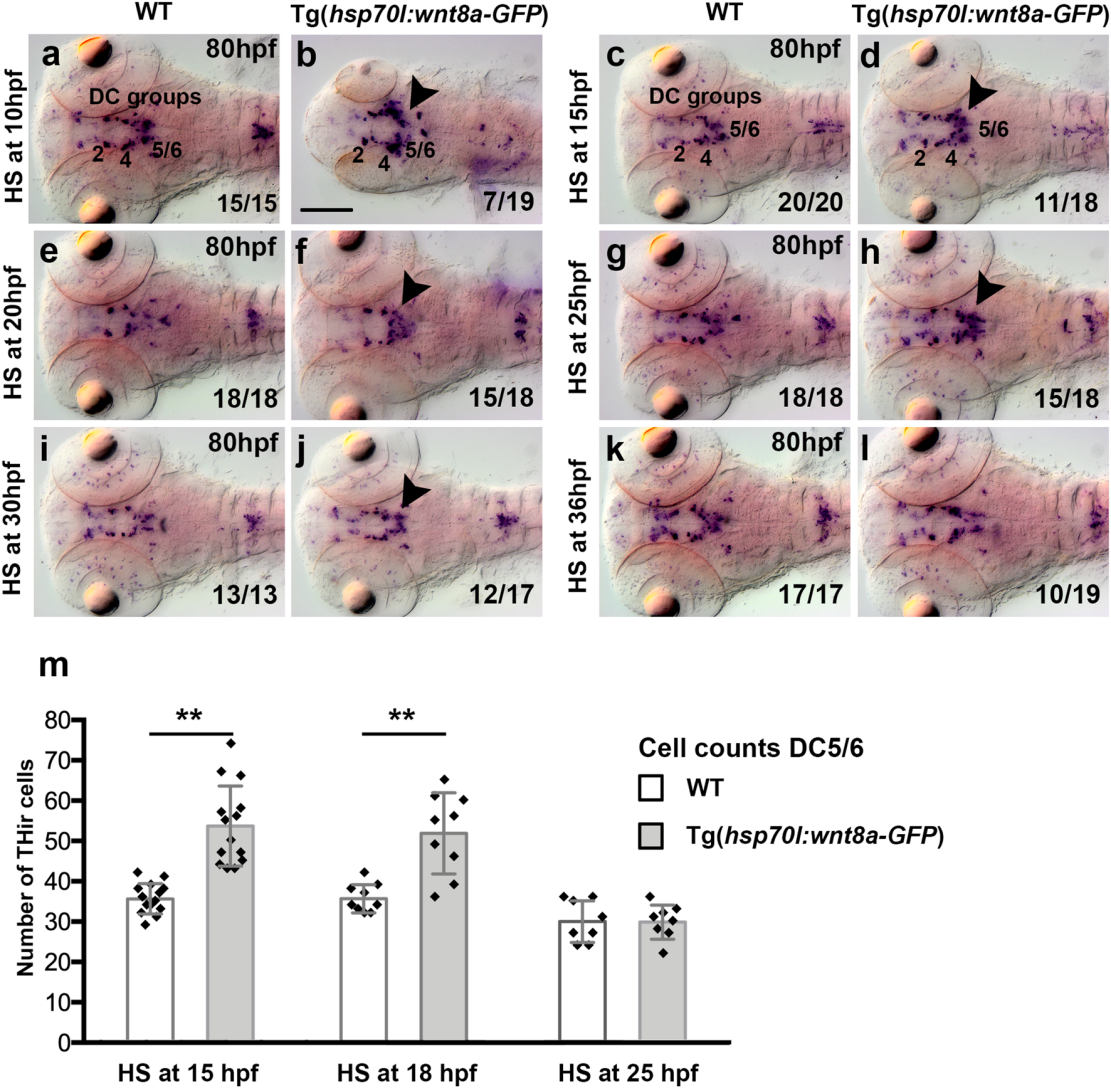
Heat shock induced Wnt8a overexpression affects hypothalamic DC5 and DC6 DA neurons in a stage dependent manner. (**a**–**l**) Expression of DA neuron marker *th* as detected by WISH in WT (**a**,**c**,**e**,**g**,**i**,**k**) and in heterozygous transgenic Tg(*hsp70l:wnt8a-GFP*) siblings (**b**,**d**,**f**,**h**,**j**,**l**) subjected to heat-shock treatments as indicated on the left and subsequently fixed at 80 hpf. Dorsal views, images are Z-projections of image stacks. The *th* expressing DC groups 2, 4, 5 and 6 are indicated in (**a**), (**b**), (**c**) and (**d**). The arrowheads in (**d**), (**f**), (**h**) and (**j**) point to an increase in *th* expression in DC5 and DC6 DA neuron groups. Scale bar in (**b**) is 100 µm for all images. Numbers N/N in bottom right corner of each image indicate number of representative phenotypes as shown in image versus total embryos analyzed for this condition. (**m**) A separate experiment was performed for quantification of TH-immunoreactive cells in WT embryos compared to heterozygous transgenic Tg(*hsp70l:wnt8a-GFP*) siblings heat shocked as indicated and fixed at 82 hpf. Bar charts show cell count numbers and the mean of TH-immunoreactive cells for each indicated DA neuron groups. Error bars depict standard deviations of the mean. Asterisks indicate significant differences compared to non-transgenic sibling control (**p = 0.001 for 15 hpf and p = 0.0009 for 18 hpf; Mann–Whitney U test; n = 14 for 15 hpf, n = 9 for 18 hpf and n = 8 for 25 hpf. Software: Prism 6.0f. from GraphPad Software Inc.).

We next analyzed the effects of genetic overexpression of Wnt/β-catenin signaling ligands or antagonists on DA neurogenesis, using heat-shock induced expression of Wnt8a from Tg(*hsp70l:wnt8a-gfp*)^w34^, Dkk1b from Tg(*hsp70l:dkk1b-gfp*)^w32^, and a dominant negative dnTcf3 (ΔTcf3) from Tg(*hsp70l:Δtcf3a-gfp*)^w26^^39–41^. To identify time windows during which the transgenic expression of Wnt/β-catenin effectors can perturb DA neurogenesis, we performed single 30 min heat shock treatments at 10 hpf, 15 hpf, 20 hpf, 25 hpf, 30 hpf or 36 hpf, and analyzed heterozygous transgenic and WT sibling embryos for *th* expression at 80 hpf (Figs. 3b and 6). Overexpression of Wnt8a led to an increase in *th* expression in DC5 and DC6 DA neuron groups following heat shocks from 10 until 25 hpf (Fig. 6a–h). Early born DC2 and DC4 *th* expressing neurons appeared unaffected by overexpression of Wnt8a when analyzed at 36 hpf and 80 hpf (Fig. 6a–h and Supplementary Fig. 5). Thus, Wnt8a overexpression during a time window when most DC5 and DC6 progenitors are still proliferative^25^ resulted in increased *th* expression and likely increased DC5/6 neuron numbers (Fig. 3b). To determine if *th* expression levels or the number of DC5/6 neurons are affected, we performed an independent experimental series using the number of TH-immunoreactive cells as readout (Fig. 6m). Overexpression of Wnt8a at 15 hpf and 18 hpf, but not at 25 hpf, increased the number of TH-immunoreactive cells of DC5 and DC6 DA neurons in Tg(*hsp70l:wnt8a-GFP*)^*w36Tg/*+^ embryos, as compared to WT siblings (Mann–Whitney U-test; p = 0.001; n = 14 for 15 hpf and p = 0.0009; n = 9 for 18 hpf; Fig. 6m). This effect was not observed by *th* WISH or TH-immunostaining in transgenic embryos heat-shocked at 30, 36, 48 or 72 hpf (Fig. 6i–l and Supplementary Fig. 6). Thus, overexpression of Wnt8a is sufficient to increase hypothalamic DC5 and DC6 DA neuron group size in a stage-specific manner. We further analyzed effects of Wnt8a overexpression on the expression of DA precursor marker genes *otpa*, *sim1a* and *neurog1*, which are the earliest transcription factors required for DA neurogenesis in the ventral diencephalon and hypothalamus^21,22,37,38^. Upon heat shocks at 15 or 20 hpf, the expression of *otpa* within the ventral diencephalon and hypothalamus appeared unaffected by Wnt8a overexpression when analyzed at 36 hpf (Supplementary Fig. 7a–d, arrowheads). When heat shocks were performed at 15 or 20 hpf, we found a mild increase in the staining intensity of *sim1a* and *neurog1* in the hypothalamus of Tg(*hsp70l:wnt8a-GFP*)^*w34Tg/*+^ embryos at 36 hpf (Supplementary Fig. 7e–l). Since Neurog1 has been linked to glutamatergic^42^ and zebrafish dopaminergic neurogenesis^38^, and since Otp-dependent DA neurons all have glutamate as second transmitter^43^, the increase in *neurog1* may correlate with an increase in DA progenitors in this region. This further supports that Wnt/β-catenin signaling acts on progenitors. To determine whether the effect of Wnt8a overexpression may be selective for DA neurons, or whether also other neuronal types developing in their proximity may be affected, we analyzed ventral diencephalic *crhb* expressing neurons, some of which also depend on Otp function^20^. The *crhb* neurons adjacent to DC2 und DC4 were unaffected by Wnt8a overexpression (Supplementary Fig. 7m–p). However, we observed an increase in *crhb* staining intensity within the pretectum (Supplementary Fig. 7m–p, arrowheads), suggesting that pretectal *crhb* neurons may be affected by Wnt/β-catenin signaling.

**Figure 7:**
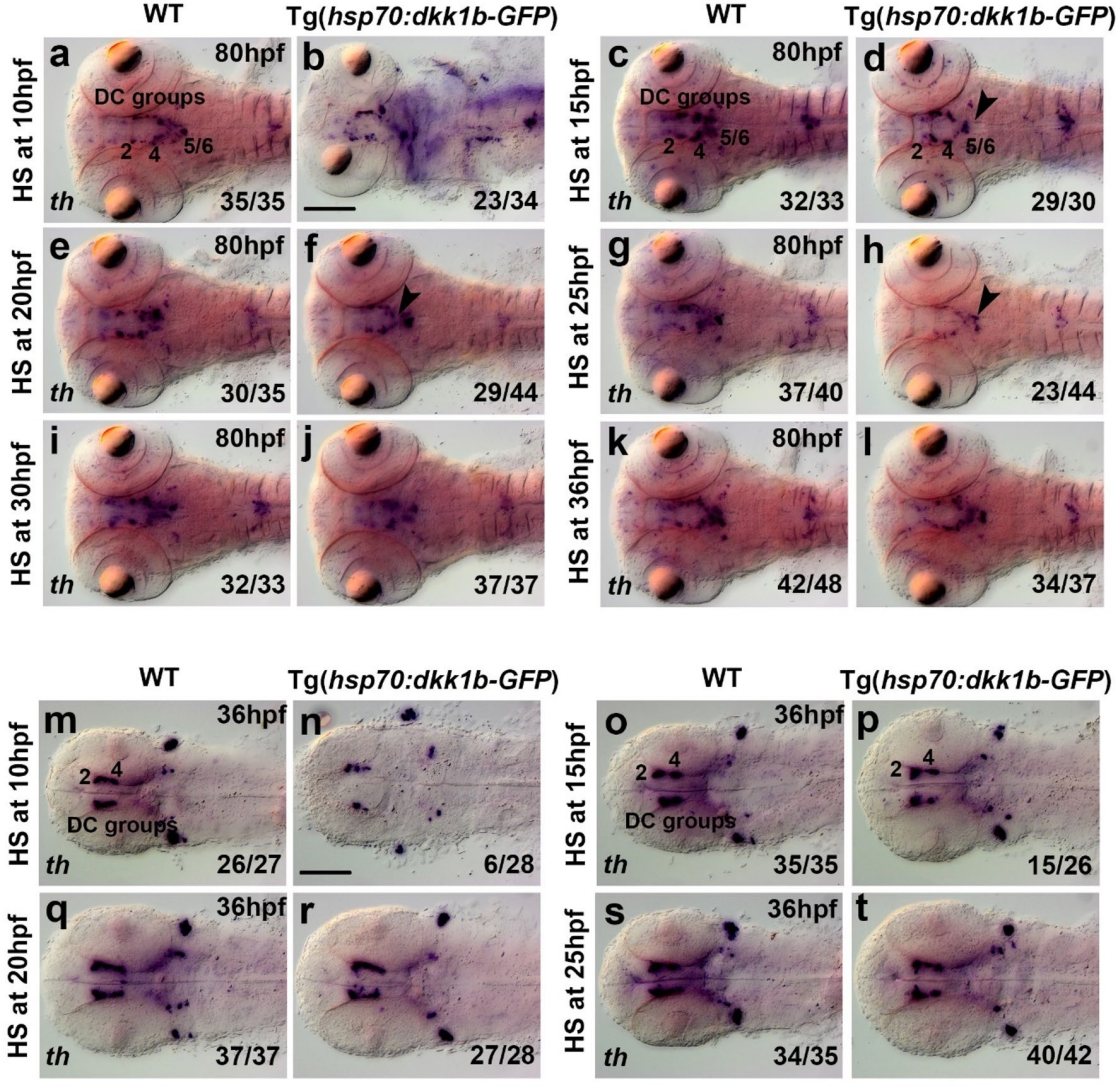
Heat shock induced Dkk1b overexpression affects hypothalamic DC5 and DC6 DA neurons in a stage dependent manner. (**a**–**s**) Expression of DA neuron marker *th* as detected by WISH in WT embryos and heterozygous transgenic Tg(*hsp70l:dkk1b-GFP*) siblings after heat-shock treatment as indicated on the left side of each pair of images, and subsequently fixed at 80 hpf (**a**–**l**) or 36 hpf (**m**–**t**). Dorsal views, images are Z-projections of image stacks. The arrowheads in (**d**), (**f**) and (**h**) point at a decrease in *th* expression in DC5 and DC6 DA neuron groups. Scale bars in (**b**) for (**a**–**l**) and in (**n**) for (**m**–**t**) are 100 µm. Numbers N/N in bottom right corner of each image indicate number of representative phenotypes as shown in image versus total embryos analyzed for this condition.

The specific expression domain of *dkk1* within the posterior tuberculum region at 36 hpf and 48 hpf (Supplementary Fig. 1n,o), suggests that Dkk1 might be involved in DA neuron development. Heat shock induced overexpression of Dkk1b between 10 and 25 hpf resulted in a reduction of *th*-expressing cells within the hypothalamic DC5 and DC6 DA groups in Tg(*hsp70l:dkk1b-GFP*)^*w32Tg/*+^ embryos analyzed at 80 hpf (Fig. 7 and Supplementary Fig. 8). Tg(*hsp70l:dkk1b-GFP*)^*w32Tg/*+^ embryos heat shocked at 10 hpf displayed strong anatomical malformations when analyzed at 80 hpf (Fig. 7b), likely due to effects on early patterning mechanisms. To determine whether early Dkk1b heat shocks may also affect DC2 and 4 DA neurons, we performed heat shocks at 10, 15, 20 and 25 hpf, and analyzed embryos directly at 36 hpf, when few DC5/6 neurons have formed (Fig. 7m–t). Following the heat shock at 10 hpf, we detected a strong reduction of DC2/4 *th*-positive neurons. We interpret this reduction as a loss of DC4 neurons, which become postmitotic slightly later (10–20 hpf) than DC2 neurons (8–12 hpf)^25^. However, since there are no molecular markers to distinguish DC2 and DC4, we cannot experimentally validate this interpretation. Heat shocks at 15 hpf or later did not affect DC2 or DC4 *th* expression. Dkk1b overexpression at either 15 or 20 hpf caused a reduction in expression of the progenitor markers *otpa* and *sim1a* within the ventral diencephalon and hypothalamus in embryos fixed at 36 hpf (Supplementary Fig. 9a–h).

**Figure 8:**
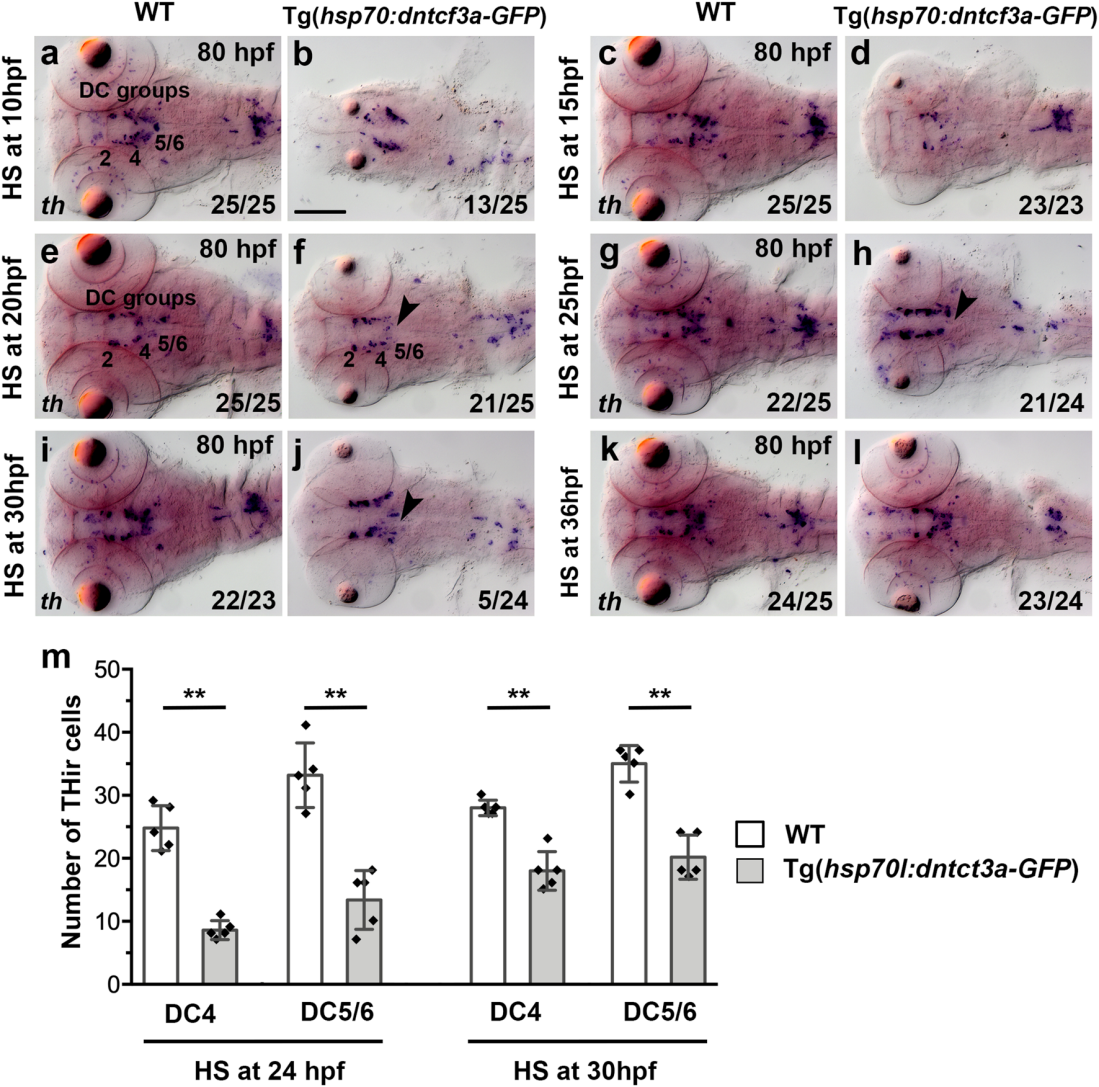
Heat-shock induced ΔTcf3 overexpression causes reduction in ventral diencephalic DC4 and hypothalamic DC5 and DC6 DA neurons. (**a**–**l**) Expression of DA neuron marker *th* as detected by WISH in WT embryos and in heterozygous transgenic Tg(*hsp70l:Δtcf3a-gfp*) [here designated Tg(*hsp70l:dntcf3-GFP*)] siblings after heat-shock treatment as indicated on the left side of each pair of images, and subsequently fixed at 80 hpf. Dorsal views, images are Z-projections of image stacks. The *th* expressing DC groups 2–6 are indicated. The arrowheads point to a decrease in *th* expression in DC5 and DC6 DA neuron groups. Scale bar in (**b**) is 100 µm for all images. Numbers N/N in bottom right corner of each image indicate number of representative phenotypes as shown in image versus total embryos analyzed for this condition. (**m**) Quantification of TH-immunoreactive cells in WT embryos compared to heterozygous transgenic Tg(*hsp70l:dntcf3-GFP*) siblings heat shocked as indicated fixed at 82 hpf. Bar charts show cell count numbers and the mean of TH-immunoreactive cells for each indicated DA neuron groups. Error bars depict standard deviations of the mean. Asterisks indicate significant differences compared with the non-transgenic sibling control (**p = 0.0079; Mann–Whitney U test; n = 5 in each condition. Software: Prism 6.0f. from GraphPad Software Inc.).

Next, we used heat shock overexpression of the dominant-negative *ΔTcf3* transcription factor^39^ to block transcriptional responses to Wnt/β-catenin signals. The overexpression of *Δtcf3* interfered most severely with normal development and caused abnormal morphology in transgenic embryos upon heat shock at any analyzed stage. Early overexpression of ΔTcf3 at 10 and 15 hpf leads to severe reduction in anterior neural tissue (Fig. 8a–d), and we excluded these stages from further analysis. Following heat shocks from 20 to 36 hpf, we see DC2 and 4 groups develop in experimental and control embryos (Fig. 8e–l). However, the stronger staining intensity in experimental embryos made it difficult to evaluate cell numbers. This effect on staining intensity may be caused by the smaller size of the ΔTcf3 embryos, as probe penetration and staining efficiency are generally superior in smaller/thinner tissue (compare for example Figs. 7a processed at 80 hpf and Fig. 7m processed at 36 hpf). To better evaluate DA cell numbers, we performed anti-TH whole mount immunofluorescence on embryos heat shocked at 24 or 30 hpf, and counted DC2, 4, 5 and 6 neurons (Fig. 8m). The cell counts revealed that, despite the apparent strong stain intensity, DC4 cell numbers are significantly reduced at both time points (Mann–Whitney U test; p = 0.0079; n = 5 for DC4 and p = 0.0079; n = 5 for DC5/6 at 24 hpf and p = 0.0079; n = 5 for DC4; p = 0.0079; n = 5 for DC5/6 at 30 hpf). DC5 and DC6 DA group sizes are reduced following heat shocks at 10 to 30 hpf (Fig. 8a–j), which is also confirmed by cell counts at 24 and 30 hpf (Fig. 8m). Thus, overexpression of ΔTcf3 affects DC5 and DC6 DA neuron development over a longer time window (10–30 hpf) compared to overexpression of Wnt8a (10–20 hpf)*,* reflecting the stronger and more immediate effect of ΔTcf3 overexpression as compared to Wnt8a overexpression on Wnt/β-catenin signaling (Fig. 3b). We observed that the effects of Wnt/β-catenin pathway manipulation on DC5 and 6 correlate with the strongest proliferation phase (red bar in Fig. 3b). While progenitor proliferation persists beyond the latest time point at which we can detect a phenotype, late effect may be too small to be detected in our WISH and immunofluorescence analysis, given that most DC5 and DC6 neurons become postmitotic before 36 hpf^25^. In summary, we conclude that Wnt/β-catenin signaling affects DC4, 5 and 6 DA progenitors predominantly during phases when they are still mitotically active.

## Discussion

In this study, we analyzed potential roles of Wnt/β-catenin signaling during development of ventral diencephalic and hypothalamic DA neuron groups in embryonic and early larval zebrafish. We focused our analysis on the Otp-dependent DC2, 4, 5 and 6 groups, which establish the zebrafish diencephalospinal dopaminergic system, homologous to the A11 system in mammals. While Wnt/β-catenin signaling mechanisms have been extensively studied in mammalian mDA neurons^10–13^, little is known about Wnt/β-catenin signaling contributions to the development of other vertebrate forebrain DA neuron clusters. Since Wnt/β-catenin signaling is required for neurogenesis in the posterior hypothalamus of zebrafish^16,26,27^, we hypothesized that Wnt/β-catenin signaling might be involved in the development of hypothalamic and posterior tubercular Otp-dependent DA neurons. Our work reveals that Otp-dependent DA neurons develop in close spatiotemporal proximity to Wnt/β-catenin pathway components expression and of Wnt/β-catenin signaling activity. Manipulation of Wnt/β-catenin signaling activity demonstrates its requirement for the development of hypothalamic DC5/6, as well as potentially for earlier forming posterior tubercular DC4 DA neurons, from cycling progenitor pools.

*wnt8b* and *wnt16* transcripts are expressed in the hypothalamic midline proliferation zone and within the posterior recess region of the hypothalamus^16,17,28^, and therefore potentially mediate Wnt/β-catenin effects on hypothalamic DA neurogenesis. We showed, that *wnt8b* is expressed in proximity of TH-immunoreactive DA neurons within the hypothalamus. We also find additional Wnt/β-catenin signaling pathway components expressed in anatomical regions of hypothalamic and ventral diencephalic DA development, including Fzd8a receptor transcripts, which has previously been shown to regulate the size of DC2/4 neural plate progenitor populations during gastrula stages^17^. In addition, we find expression of the antagonists *sfrp5* (24–36 hpf) and *dkk1* (36–48 hpf) in proximity to DC2,4–6 DA neurons, suggesting a potential involvement in their development. In mammals, Dkk1 is involved in neuronal differentiation, particularly in mDA progenitors both in vitro and in vivo^44,45^. In this study, we demonstrated that the heat shock induced overexpression of Dkk1b leads to a temporally restricted effect on DC5 and DC6 neurons only during segmentation stages, correlating with stages in which *dkk1* shows a small dynamic expression domain in the forebrain^29^. Interestingly, other secreted Wnt/β-catenin signaling antagonists are also expressed during segmentation stages within the ventral forebrain. At 15 hpf the secreted antagonists *frzb*, as well as *sfrp1a* and *sfrp5* are broadly expressed within the ventral forebrain and presumptive hypothalamus^46,47^. However, functional analysis of both Wnt/β-catenin ligands and antagonists during embryonic and early larval development of the A11-type DA neurons of the diencephalospinal system have still been missing.

Using two different reporter lines for Wnt/β-catenin-dependent transcriptional activity, we show that posterior tubercular and hypothalamic DA neurons of the DC2,4–6 groups differentiate in close proximity to cells that show active Wnt/β-catenin dependent transcription. However, we found that Wnt/β-catenin signaling reporter expression is absent from post-mitotic *th* expressing DC2,4–6 DA neurons. The stability of GFP in cells (half-life typically several hrs) suggests that Wnt/β-catenin-dependent transcriptional activity is also likely absent from postmitotic progenitors that have not yet started to express *th.* These findings are in line with previous reports describing the absence of Wnt/β-catenin signaling reporter expression in TH-immunoreactive DA cells and committed progenitor cells in the zebrafish hypothalamus at 32 hpf^26,27^.

In contrast to DC2,4–6 DA neurons, we observed that in another hypothalamic DA neuron populations, DC3, a small number of DA neurons express the Wnt/β-catenin reporter. DC3 may correlate to mammalian A14 neurons, and is located close to the midline in the zebrafish hypothalamus in what likely corresponds to mammalian mamillary/retromamillary areas^19^. While we did not investigate this group in detail, the small number of GFP positive neurons would be consistent with a model in which the mature DC3 DA neurons are not transcriptionally responding to Wnt/β-catenin signaling, but DC3 progenitors may express the Wnt/β-catenin reporter, and some GFP persists into early DC3 neurons. DC3 neurons form over an extended developmental time, while DC2/4 neurons mature in a rather small and early time window^25^, thus chances to detect GFP positive early DC3 neurons are higher. The absence of Wnt/β-catenin reporter GFP signal from mature DC2,4–6 DA neurons does not exclude that Wnt/β-catenin signaling may be active in their progenitor populations: in fact, we detect Wnt/β-catenin reporter activity in neuroepithelial cells at postgastrula stage adjacent to DA neurons, and on the second day of development in cells medial to the DC2,4–6 groups. This reporter expression is consistent with the location of neural stem and progenitor cells at the midline ventricular wall of the ventral diencephalon and hypothalamus, some of which may be stem cells developing into DC5/6 progenitors. One additional hypothalamic DA group, DC7, which develops in the caudalmost hypothalamus in the posterior recess region (likely tuberal hypothalamus) has been shown to develop in proximity of Wnt/β-catenin reporter activity, and for adjacent *th2* expressing DA neurons of the posterior recess, it has been demonstrated by lineage tracing experiments that they arise from radial glia populations that express Wnt/β-catenin reporter activity^15^. It was recently shown that *th2* DA neurons in the caudal hypothalamus were unaffected in *lef1* mutants^48^, however, since Lef1 and Tcf1 act at least partially redundant in many systems^49^, this does not exclude a role of Wnt/β-catenin signaling in DC7 development.

We performed a temporally controlled analysis of the impact of Wnt/β-catenin signaling activity on DC2,4–6 DA development using pharmacological inhibition and activation of Wnt/β-catenin signaling, as well as genetic overexpression of a prototypical canonical ligand (Wnt8a) and Wnt/β-catenin pathway antagonists (Dkk1 and ΔTcf3). Our results support a model for Wnt/β-catenin signaling activity during DC2,4–6 neurogenesis. At late gastrula stages prior to 10 hpf, strong activation or inhibition of Wnt/β-catenin signaling interferes with global anterioposterior patterning in the neural plate^17^, which affects brain morphogenesis and makes it difficult to assess DA neuron numbers in specific DA groups. At postgastrula stages into the second day of development (about 35 hpf), when DA neurogenesis derives from proliferating progenitor populations^25^, enhanced Wnt/β-catenin activity results in development of an increased number of DC4-6 neurons, while inhibition of Wnt/β-catenin signaling reduces the number of DA neurons in these groups. Previous birth dating experiments on DA neurons in the zebrafish forebrain revealed that more than 50% of DC5 and DC6 progenitors are still in a proliferative state between 15 and 24 hpf^25^. Thus, Wnt/β-catenin signaling modulation elicited an effect on DA group size specifically at stages when progenitors are cycling. Finally, in embryos older than 48 hpf, upon Wnt/β-catenin manipulation, we could not observe significant changes in DA numbers anymore. However, we cannot exclude an effect of Wnt/β-catenin signaling on late embryonic progenitors since DC5/6 DA neurogenesis slows down, such that a significant change in the total number of DA neurons in each cluster is difficult to observe.

To confirm whether DC2,4–6 progenitors were affected by Wnt/β-catenin signaling, we analyzed expression of their markers *otpa* and *neurog1*. Interestingly, pharmacological activation of Wnt/β-catenin signaling increased, while its inhibition decreased the expression of *otpa* and *neurog1* within the hypothalamus. These findings support our interpretation that Wnt/β-catenin signaling acts on DC2,4–6 progenitors. In accordance, it has been shown that Wnt8b signals stimulate progenitor proliferation in the zebrafish posterior hypothalamus via Lef1^16,26^. At 32 hpf, Wnt/β-catenin signaling is active in non-committed, proliferative progenitors in the hypothalamic posterior recess and the pathway positively regulates proliferation and expansion of these cells^26^. *Lef1* mutant zebrafish show proliferation defects in the hypothalamus as indicated by decreased size of its hypothalamic tissue and by a decrease in the number of proliferative progenitors in the posterior recess region^26^. Therefore, the changes in the hypothalamic DC5 and DC6 DA neuron groups may be mediated by Wnt/β-catenin signaling acting on progenitor proliferation in the hypothalamus.

The earliest differentiating DA neurons of DC2 undergo neurogenesis directly from neural plate derived precursor cells^25^. Previous work revealed that early Wnt/β-catenin signaling active during gastrula stage neural plate patterning restricts the size of the DC2 neuron group within the neural plate^17^. Specifically, Russek-Blum et al. showed that overexpression of Dkk1 during gastrulation by mRNA injections into the zygote, or by heat shock expression of Dkk1 at 4 or 6 hpf, as well as by Morpholino knockdown of *wnt8b*, significantly increased DC2/4 neuron numbers. Thus, when Wnt/β-catenin signaling activity is reduced during gastrulation, Russek-Blum et al. observe that more DC2/4 DA neurons form, indicating that active Wnt/β-catenin signaling at gastrula stages negatively affects the DA progenitor pool. We observe the opposite effect of Wnt/β-catenin signaling on DA neuron development during post-gastrula stages, and show that enhanced Wnt/β-catenin activity during somitogenesis between 10 and 30 hpf increases the number of DC4-6 DA neurons. The requirement for Wnt/ß-catenin signaling in late DA precursor pool expansion may extend even later in development, as suppression of Wnt/β-catenin signaling activity negatively affects DA neuron numbers in our experiments as late as 35 hpf. Accordingly, we did not observe any effects of altered Wnt/β-catenin signaling activity from 10 hpf onwards on DC2 DA neuron groups, which mostly become postmitotic before 12 hpf. Together, the work by Russek-Blum et al. and our findings suggest that Otp-dependent, A11-type DA neurons may be affected by fundamentally different Wnt/β-catenin signaling mechanisms during neural plate patterning compared to later development of these DC DA neuronal groups from hypothalamic and ventral diencephalic proliferation zones.

In summary, our study reveals an important role of Wnt/β-catenin signaling activity during hypothalamic DA neuron development from proliferative progenitor pools. Our data suggest that Wnt/β-catenin signaling may promote the expansion of proliferating hypothalamic progenitor pools to regulate the size of the early posterior tubercular DC4 as well as the later developing hypothalamic DC5 and DC6 DA neuron groups in a stage-specific manner. Multiple roles of Wnt/β-catenin signaling in hypothalamic development have also been uncovered in mammalian systems^50,51^. It will be interesting to learn how Wnt/β-catenin signaling is integrated with other signaling pathways that have recently been shown to be active in DA progenitors of the zebrafish hypothalamus, including Shh signaling, Ghrelin signaling, as well as microRNAs^52–54^. In zebrafish, loss of function of *ghrelin* leads to a downregulation of Wnt/β-catenin genes and concomitantly to a reduction of DA neurons ^54^. Interestingly, pharmacological activation of the Wnt/β-catenin signaling pathway rescues the DA neuron loss in *ghrelin* mutants, demonstrating a functional link between Ghrelin and Wnt/β-catenin signaling in DA neurogenesis. Furthermore, microRNA miR-7 controls Wnt/β-catenin signaling activity and thereby regulates hypothalamic DA neurogenesis in zebrafish^53^. While mutations affecting Shh signaling do not impair posterior tubercular DC2/4 DA development in the embryo^55^, during later development posterior tubercular DA neurons have been reported to arise from Shh expressing cells^56^. It has not escaped our attention that there are interesting similarities in Wnt/β-catenin and Shh requirements in mammalian mes-diencephalic DA development^5,12,57^, mammalian hypothalamic tanycytes^58^, and zebrafish ventral hypothalamic DA development.

## Material and methods

### Zebrafish husbandry and strains

Zebrafish care and breeding were performed under standard conditions as described in *The Zebrafish Book*^59^ in glass or polycarbonate tanks (Aqua Schwarz GmbH, Göttingen) at 26–28 °C, three water changes per hour, and on a 14/10 h light/dark cycle with the lights on at 8:30 AM. Zebrafish embryos were maintained at 28.5 °C in 1 × E3 medium (5 mM NaCl, 0.17 mM KCl, 0.33 mM CaCl2, 0.33 mM MgSO4) containing 0.2 mM phenylthiourea (1 × PTU) to prevent pigmentation. Embryos were staged according to^60^.

The zebrafish AB/TL strain was used as wildtype zebrafish. The following transgenic reporter lines were used: Tg(*top:dGFP*)^w25^ ^31^, and Tg(*7xtcf-Xla.siam:gfp*)^ia4^. Genetic gain of function experiments used the following transgenic lines: Tg(*hsp70l:wnt8a-gfp*)^w34Tg ^^41^, Tg(*hsp70l:dkk1b-gfp*)^w32Tg^^40^, and Tg(*hsp70l:Δtcf3a-gfp*)^w26Tg^^39^.

### Ethics statement

All animal experiments were approved by state authorities (Ethics Commission for Animal Experiments of the Regional Council of the State of Baden-Württemberg in Freiburg, Department 35 [Regierungspraesidium Freiburg], Germany; permits 35-9185.81/G-16/123, 35-9185.81/G-15/44), and all experiments performed in accordance with German animal law and regulations (Tierschutzgesetz TierSchG version 18 May 2006 with all modifications included until 10 August 2021). Data availability statement: all data are included in the figures and supplementary materials; primary data microscopic images are available upon request (driever@biologie.uni-freiburg.de). The study is reported in accordance with ARRIVE guidelines (https://arriveguidelines.org). The authors have declared that no competing interests exist.

### Small molecule treatments

The following small molecule antagonists of the Wnt/β-catenin signaling pathway were used: IWR-1^33^ obtained from Sigma (#I0161) and XAV939^34^ obtained from Maybridge (#RF03920). Additionally, the canonical Wnt agonist 6-bromoindirubin-3′-oxime, herein referred to as BIO^35^ obtained from Sigma (#B1686) was used. All small molecule compounds were dissolved in DMSO (99.59%, PanReac AppliChem #A3672) at 10 mM stock concentration. For experiments, the 10 mM stock was diluted in 1 × E3/1 × PTU medium to obtain the desired working concentrations. The antagonist IWR-1 was used at 20 µM (based on active concentration of 10 µM demonstrated for zebrafish by Chen et al.^33^) and the antagonist XAV939 was used at 10 µM (based on active concentration of 5 µM demonstrated for zebrafish by Huang et al.^34^). The agonist BIO was used at 10 µM (based on active concentrations range between 5 and 50 µM on Xenopus embryos by Meijer et al.^35^, and 10 µM on zebrafish embryos by Ref.^61^). Stage matched embryos obtained from separate AB/TL incrosses were pooled and incubated for different time windows in 1 × E3/1 × PTU medium containing small molecule compounds at 28.5 °C. Compound solution was removed by washing 5 times in 1 × E3 medium for 5 min. at room temperature. Embryos were subsequently incubated at 28.5 °C in 1 × E3/1 × PTU until the desired developmental stage was reached. The schematic overview in Fig. 3 was generated using Adobe Photoshop CS6 software.

### EdU pulse labeling

Proliferation of hypothalamic neural stem and progenitor cells was determined by 5-ethynyl-2′-deoxyuridine (EdU) pulse labeling^25^. Zebrafish embryos at 34 hpf were incubated for 20 min in 10 mM EdU/15% DMSO on ice. The pulse was stopped by removing the EdU and embryos were washed 3 times in 1 × E3 medium. Embryos were incubated in 1 × E3/1 × PTU at 28.5 °C for 2 h. After the chase, embryos were fixed in 4% PFA for 4 h at RT. EdU incorporation was detected by click-it chemistry mediated marking of incorporated EdU with Alexa Fluor488 azide, using the Click-It EdU Imaging Kit (Invitrogen/ThermoFisher).

### Heat shock treatments

For heat-shock induced genetic overexpression experiments, three transgenic zebrafish lines were used: Tg(*hsp70l:wnt8a-gfp*)^w34Tg^^41^, Tg(*hsp70l:dkk1b-gfp*)^w32Tg^^40^, and Tg(*hsp70l:Δtcf3a-gfp*)^w26Tg^^39^. We reasoned that differences in expressed transgene levels might affect phenotypes differently. To keep transgene levels similar, we used heterozygous transgenic embryos for all experiments. Therefore, only embryos derived from outcrosses of heterozygous transgenic fish to AB/TL fish were used. Prior to each heat-shock treatment medium was replaced with 1 × E3/1 × PTU solution preheated to 40 °C. Heat-shock treatments were performed for 30 min at 39 °C in a water bath. After heat-shock treatment embryos were transferred into fresh 1 × E3/1 × PTU medium and incubated at 28.5 °C. Two hours post heat shock, treated embryos were sorted for transgene expression into GFP-positive heterozygous transgenic embryos, and GFP-negative non-transgenic embryos. The non-transgenic siblings served as control embryos. Embryos were incubated until fixation in 1 × E3/1 × PTU at 28.5 °C.

### Whole mount chromogenic and fluorescent in situ hybridization

Zebrafish embryos were fixed in 4% PFA at the desired developmental stages. Alkaline Phosphatase based colorimetric whole mount in situ hybridization (WISH) was performed as previously described^62,63^. Digoxygenin (DIG) labeled antisense riboprobes were in vitro transcribed from linearized plasmids along with DIG labeling mix (Roche). The following DIG labeled antisense riboprobes were used: *crhb*^21^, *dkk1*^29^, *fzd8a*^64^, *fzd8b*^64^, *otpa*^22^, *sfrp5*^47^, *neurog1*^65^, *sim1a*^21^, *th*^62^, *uts1*^36^, *vip*^36^ and *wnt8b*^66^.

A 992 bp cDNA fragment of *axin2* (NCBI GenBank AF387812) was amplified by RT-PCR using RNA isolated from WT zebrafish embryos at 24 hpf. For PCR amplification the following primers were used: *axin2* forward primer 5′GGAGAGGAGGTGAACATGGA3′ and *axin2* reverse primer 5′ATCATCACGAATGCTGGTCA3′. The amplified *axin2* cDNA fragment was cloned into the pCRII-TOPO vector (Invitrogen). For synthesis of a DIG-labeled antisense riboprobe, the pCRII-*axin2* vector was linearized with XhoI (NEB) and *in-vitro* transcribed with a SP6 RNA polymerase (Roche).

Tyramide signal amplification (TSA)-based fluorescent in situ hybridization (FISH) for *wnt8b, sox2*^67^ or *sox3*^67^ combined with fluorescent immunohistochemistry for Tyrosine Hydroxylase (TH) or Sox2 was performed as described^68^.

### Whole mount immunofluorescence

Whole mount immunofluorescence was performed as described in Ref.^69^. Following FISH, a rabbit polyclonal anti-TH primary antibody^22^ was used at 1:500 dilution and detected with an anti-rabbit Alexa555-conjugated secondary antibody (2 µg/ml, Life Technologies A-21428). For double FIHC (Tg(*top:dGFP*)^w25^ and Tg(*7xtcf-Xla.siam:gfp*)^ia4^ embryos), a chicken anti-GFP antibody (5 µg/ml; Invitrogen) was combined with a polyclonal rabbit anti-TH antibody^22^. Primary antibodies were detected with an anti-chicken Alexa488 antibody (2 µg/ml; Life Technologies A11039) and an anti-rabbit Alexa555 antibody (2 µg/ml, Life Technologies A21430). Following FISH for *sox2* and *sox3*, primary mouse-anti-Sox2 antibody (2.5 µg/ml, Abcam, ab171380) was used and subsequently detected using an anti-mouse-Alexa633 (2 µg/ml, Life Technologies A21050) antibody. Following EdU detection, the Sox2 antibody was detected using an anti-mouse-Alexa555 secondary antibody (2 µg/ml, Life Technologies A21422).

### TUNEL assay

Detection of apoptotic cell death by TUNEL staining was performed as described^63^ using the Chemicon ApopTag In Situ Apoptosis Detection Kit (Millipore Bioscience Research).

### Microscopy and image analysis

Transmitted light images of WISH stained embryos were recorded using an Axioskop compound microscope (Carl Zeiss) with DIC optics and an AxioCam MRc digital camera (Carl Zeiss).

Fluorescently immunolabeled embryos were recorded as confocal stacks. Confocal imaging was performed using a Carl Zeiss LSM 510 upright laser scanning microscope. The LD LCl Plan-Achromat 25x-objective (NA = 0.8) with glycerol as an immersion medium was used. The 488 nm, the 543 nm and the 633 nm laser lines were used for confocal microscopy. The pinhole sizes were adjusted to 1 Airy Unit.

For cell counts, fluorescent light images were captured using a Carl Zeiss Axio Examiner and an AxioCam MRc digital camera. The Zeiss Zen Blue software was used for cell number quantification in z-stacks. The channel was converted to grayscale to enhance contrast. The ZEISS ZEN Event Marker Tool was used to manually mark each cell and its coordinates in its x, y and z plane. Frequent retracking of each marked cell through the z-stack was done to make sure that every cell was counted once only. For the cell counts of Sox2 and EdU labelled proliferative stem cells the Fiji 2.0.0-rc-69 software with the Cell Counter plugin was used to manually mark each cell in x and y plane. Substacks of 37.5 µm were analyzed with cells counted in image planes 7.5 µm distant from each other to avoid recounting.

For differential interference contrast (DIC) transmitted light images of embryos recorded as z-stacks, as indicated in figure legends minimum intensity z-projections were generated in ImageJ using the integrated Z-projection tool (ImageJ version 1.52 s, downloaded from https://imagej.nih.gov/ij/download.html). The ZEISS ZEN Blue 3.2 Software was used to generate maximum intensity projections of selected focal planes in confocal immunofluorescence stacks. Levels were linearly adjusted by histogram clipping using Adobe Photoshop CS6. Figures were assembled using Adobe Photoshop CS6 or Adobe Photoshop 22.3.0.

## Supplementary Information

Supplementary Video 1.

Supplementary Video 2.

Supplementary Video 3.

Supplementary Video 4.

Supplementary Video 5.

Supplementary Video 6.

Supplementary Video 7.

Supplementary Information.
