# Supplementary material for: Wnt/β-catenin signaling promotes neurogenesis in the diencephalospinal dopaminergic system of embryonic zebrafish
Source: Sci Rep. 2022 Jan 19;12:1030. doi: 10.1038/s41598-022-04833-8 (PMC8770493; doi:10.1038/s41598-022-04833-8)
Supplement: Supplementary file 8 — Supplementary Information. [file 41598_2022_4833_MOESM8_ESM.pdf]

Supplementary Materials

Wnt/ß-catenin signaling promotes neurogenesis in the diencephalospinal dopaminergic system of embryonic zebrafish.

Markus Westphal, Paolo Panza, Edda Kastenhuber, Johanna Wehrle and Wolfgang Driever

Supplementary Table 1

Supplementary Figures 1 through 8

Supplementary Videos 1 through 7

Supplementary References

	Gene	Expression	Reference
**Wnt/ß-catenin ligands**	*wnt7*	rostral diencephalon	[1]
	wnt8b	diencephalon, hypothalamus	[2,3]
	wnt11	ventral diencephalon, hypothalamus	previously *wnt11r* [2]
	wnt16	ventral diencephalon; hypothalamus	[2]
**Frizzled receptors**	*fzd7a*	ventricular zone, hypothalamus	http://zfin.org/ZDB-GENE-990415-223
	fzd7b	ventricular zone, hypothalamus	[4]
	fzd8a	subpallium, diencephalon, hypothalamus	[5]
	fzd8b	subpallium, diencephalon, hypothalamus	[5]
	fzd8c	diencephalon	[6]
**Extracellular Wnt inhibitors**	*sfrp1a*	subpallium, thalamus, hypothalamus	[7]
	sfrp5	subpallium, thalamus, hypothalamus	[8]
	wif1	hypothalamus, ventral midbrain	[9]
	dkk1	neuroectoderm, ventral diencephalon (after 24 hpf)	[10] this study

Supplementary Table 1: Overview of zebrafish *wnt*, *frizzled* and Wnt extracellular inhibitor gene expression in the developing brain. The table only lists those genes for which expression in regions of DA cell groups might suggest a role in development of Otp-dependent DA neurons.

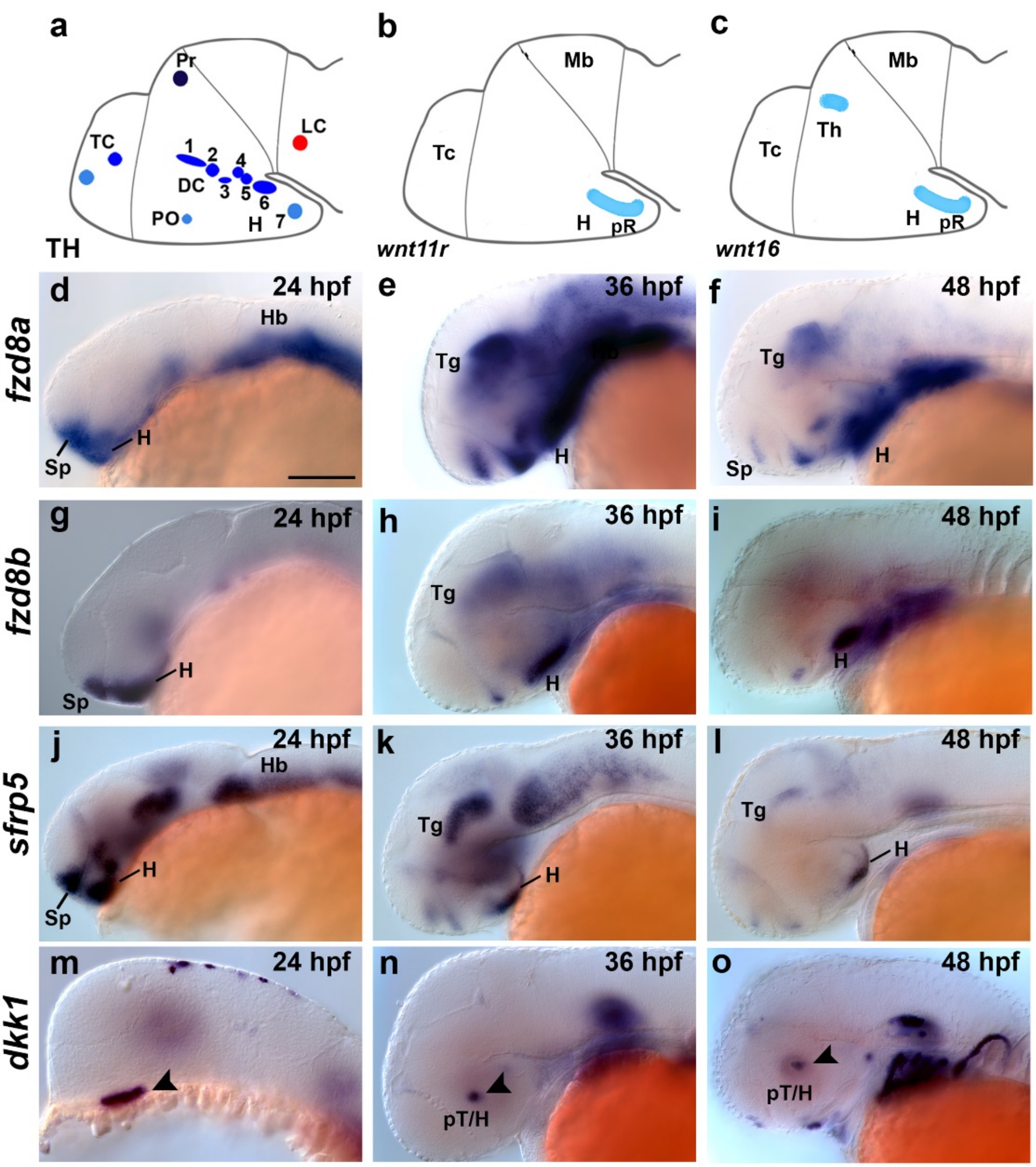

Supplementary Figure 1: Expression of selected Wnt/ß-catenin signaling ligands, receptors and extracellular antagonist genes in the brain of zebrafish embryos.
(**a-c**) Schematic drawings of zebrafish embryonic brains in lateral views at 72 hpf. (a) Distribution of DA neurons (blue dots; taken from [11]). DA neuron groups develop in the zebrafish forebrain in the telencephalon (Tc), pretectum (Pr), preoptic area (PO) as well as the ventral diencephalon (DC) and hypothalamus (H). The scheme also shows the location of noradrenergic neurons of locus coeruleus (red). (b) *wnt11* expression and (c) *wnt16* expression. Expression of *wnt11* (b, previously named *wnt11r*) and *wnt16* (c) depicted based on [2]. (**d-o**) Expression analysis of *frz8a* (d-f), *frz8b* (g-i), *sfrp5* (j-l) and *dkk1* (m-o) in zebrafish embryos at indicated stages as detected by WISH. Lateral views of heads of embryos, images show Z-projections of image stacks. Arrow in (m) points at the prechordal mesoderm. Scale bar in (**d**) is 100 µm for all images. Abbreviations: DC: diencephalon; H: hypothalamus; Hb: hindbrain; LC: locus coeruleus; Mb: midbrain; PO: preoptic region; Pr: pretctum; pR: posterior recess; pT: posterior tuberculum; Sp: subpallium; Tc: telencephalon; Tg: tegmentum; Th: thalamus. Graphics and figure assembled using Photoshop CS6 (Adobe Corp.).

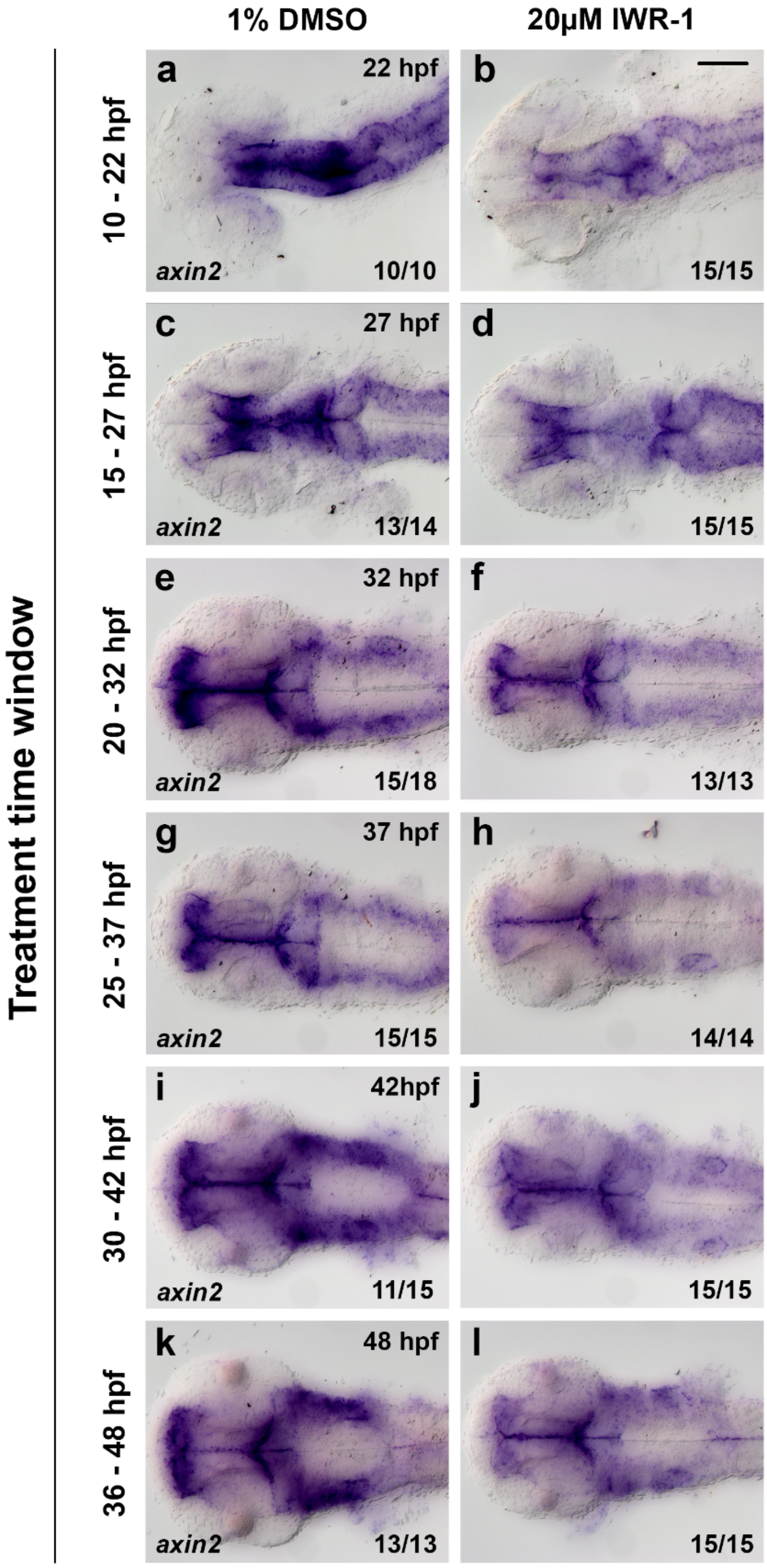

Supplementary Figure 2: Effects of IWR-1 treatment on expression of Wnt/ß-catenin signaling target gene *axin2*.

(a-l) Expression of the Wnt/ß-catenin signaling target gene *axin2* by WISH in embryos after IWR-1 or DMSO control treatments during indicated time windows (left) and fixed at time points indicated in images top right corners. Dorsal views of heads of larvae, images show Z-projections of image stacks. Scale bar in (b) is 100 µm for all images. Numbers N/N indicate number of representative phenotypes as shown in image versus total embryos analyzed for this condition.

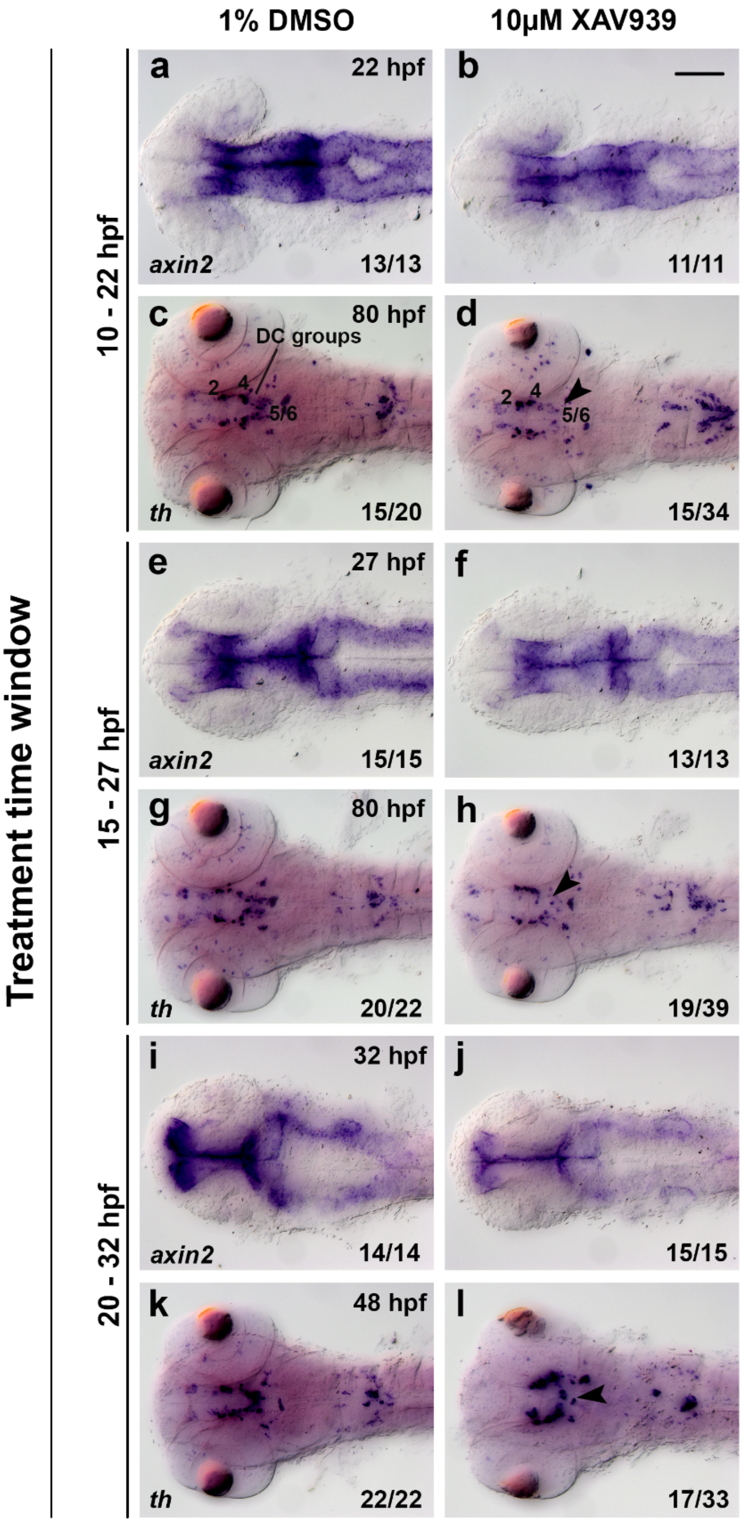

Supplementary Figure 3: Effects of treatments with the Wnt/ß-catenin signaling antagonist XAV939 on WNT target gene *axin2 a*nd DA neurons.

(a-l) Expression of Wnt/ß-catenin signaling target gene *axin2 (*a,b,e,f,I,j) and the DA neuron marker *th* (c,d,g,h,k,l) analyzed by WISH following treatments with DMSO or 10 µM XAV939 during time windows indicated at left. Fixation time point as indicated in images. The *th* expressing DC groups 2-6 are indicated in (c) and (d). The arrowhead in (d), (h) and (l) point to decreased *th* expression in DC5/6 group. Dorsal views of heads of larvae, images show Z-projections of image stacks. Scale bar in (b) is 100 µm for all images. Numbers N/N indicate number of representative phenotypes as shown in image versus total embryos analyzed for this condition.

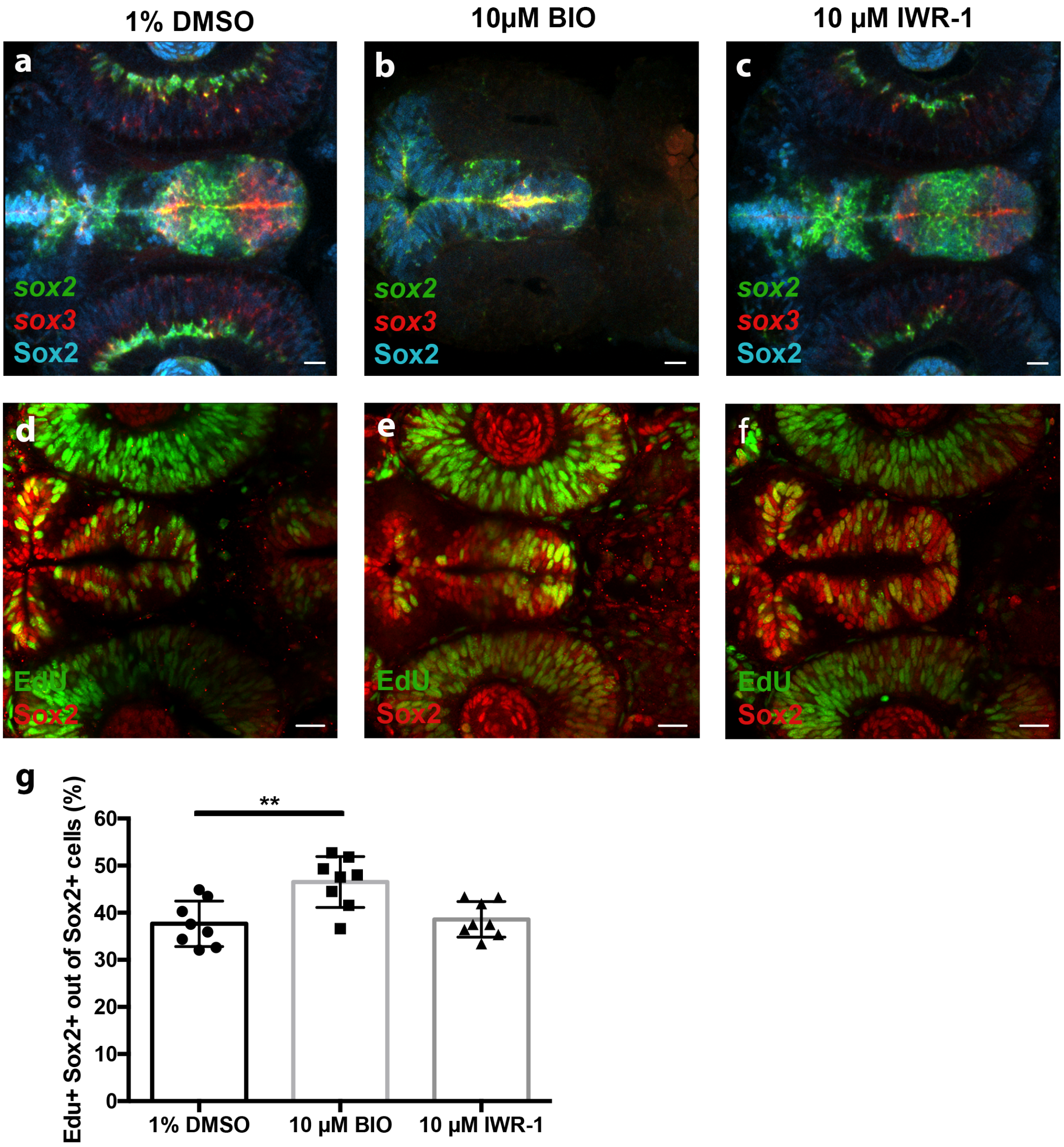

Supplementary Figure 4: Pharmacological activation of Wnt/ß-catenin signaling affects proliferation of stem and/or progenitor cells in the hypothalamus.

(**a-c**) Co-expression analysis of *sox2* and *sox3* by fluorescent *in situ* hybridization and of Sox2 immunoreactive cells by immunohistochemistry. (**d-f**) Detection of EdU incorporation and Sox2 immunoreactivity by immunohistochemistry in WT embryos treated with (a,d) 1% DMSO (control), (b, e) 10 µM BIO, or (c,f) 10 µM IWR-1. Embryos were incubated in compound solution or DMSO (a-c) between 16 hpf and 42 hpf, fixed at 48 hpf or (d-f) incubated between 15 hpf and 34 hpf, EdU pulsed at 34 hpf and fixed at 36 hpf. Dorsal views, scale bar is 20 µm for all images. (**g**) Quantification of the EdU and Sox2 double positive cells as percentage of the total number of Sox2-positive cells. For 4 experimental embryos each, the cells in left and right half hypothalamus were counted separately to obtain 8 data points. Average count of Sox2 positive cells per half hypothalamus were 348 for DMSO control, 259 for BIO and 370 for IWR-1 treated embryos. Error bars depict standard deviations of the mean. Asterisks indicate significant difference of treated embryos compared to control embryos ($p=0.0023$; one-way ANOVA, $n=8$ for each condition. Software: Prism 6.0f from GraphPad Software Inc.).

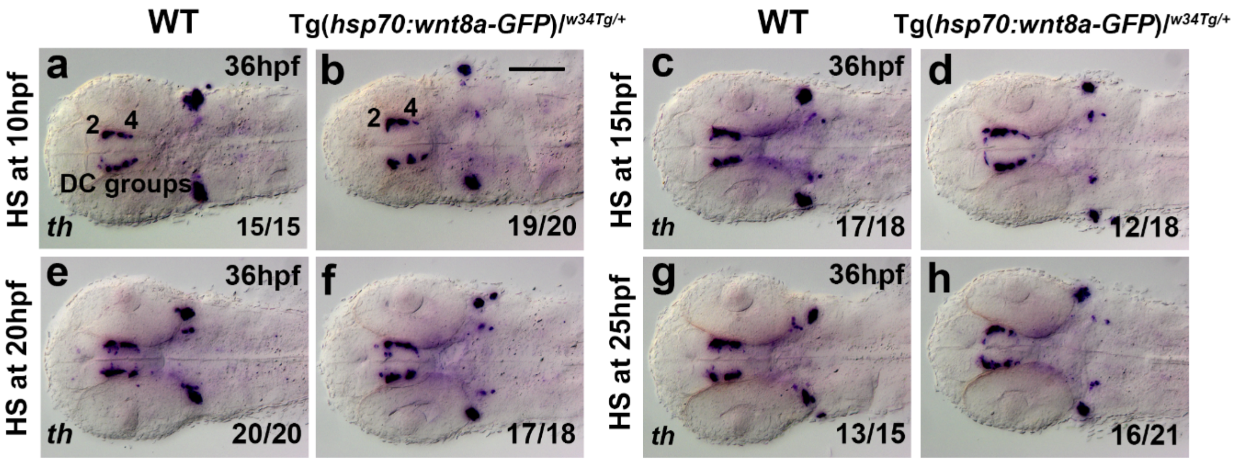

Supplementary Figure 5: Effects of heat shock Wnt8a overexpression on ventral diencephalic DC2 and DC4 DA neurons analyzed at 36 hpf.

(a-h) Expression of the DA neuron marker *th* as detected by WISH in WT embryos (a, c, e, g) and in heterozygous transgenic Tg(*hsp70l:wnt8a-GFP*)$^{w34Tg/+}$ siblings (b, d, f, h) after heat-shock treatments. Time point of the heat-shock treatment as indicated on the left side of each row. Embryos were fixed at 36 hpf. DA neuron DC2 and DC4 groups are indicated in (a) and (b). Dorsal views of heads of larvae, images generated from Z-projections of image stacks. Scale bar in (b) is 100 μm for all images. Numbers N/N indicate number of representative phenotypes as shown in image versus total embryos analyzed for this condition.

a

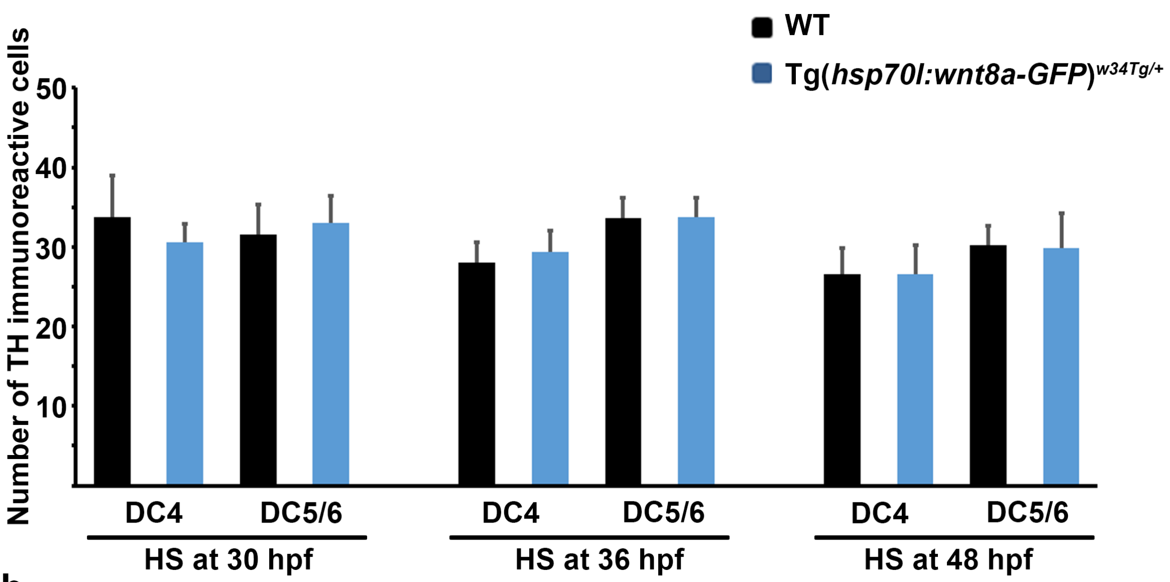

b

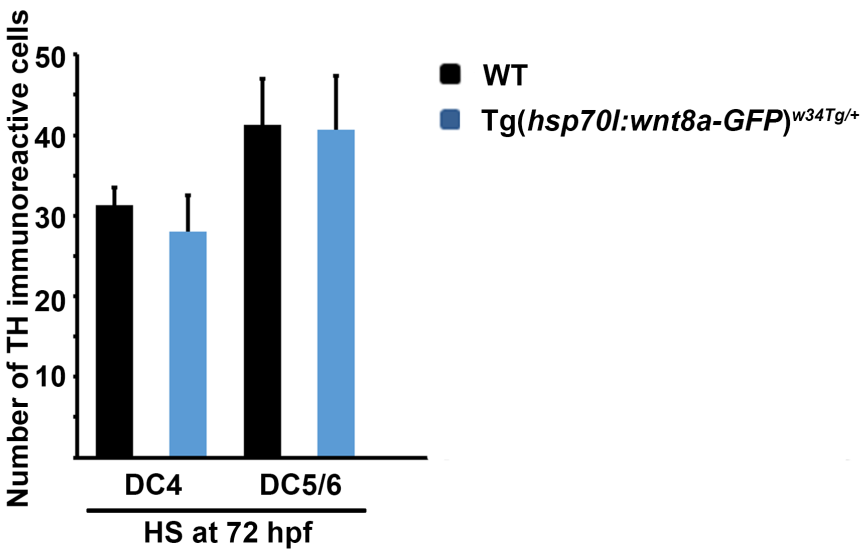

Supplementary Figure 6: Effects during the second and third day of development of heat-shock induced Wnt8a overexpression on TH-immunoreactive cell numbers.

(a,b) Quantification of TH-immunoreactive cells in WT embryos compared to heterozygous transgenic Tg(*hsp70l:wnt8a-GFP*)$^{34Tg/+}$ siblings heat shocked at either 30 hpf, 36 hpf or 48 hpf, and fixed at 82 hpf (a), or heat-shocked at 72 hpf and fixed at 96 hpf (b). Bar charts show the mean cell count numbers of TH-immunoreactive cells for each indicated DA neuron groups. Error bars depict standard deviations of the mean (Mann-Whitney U test; p> 0.05 in each condition; n=5 in each condition). Software: Microsoft Excel Version 16.54.

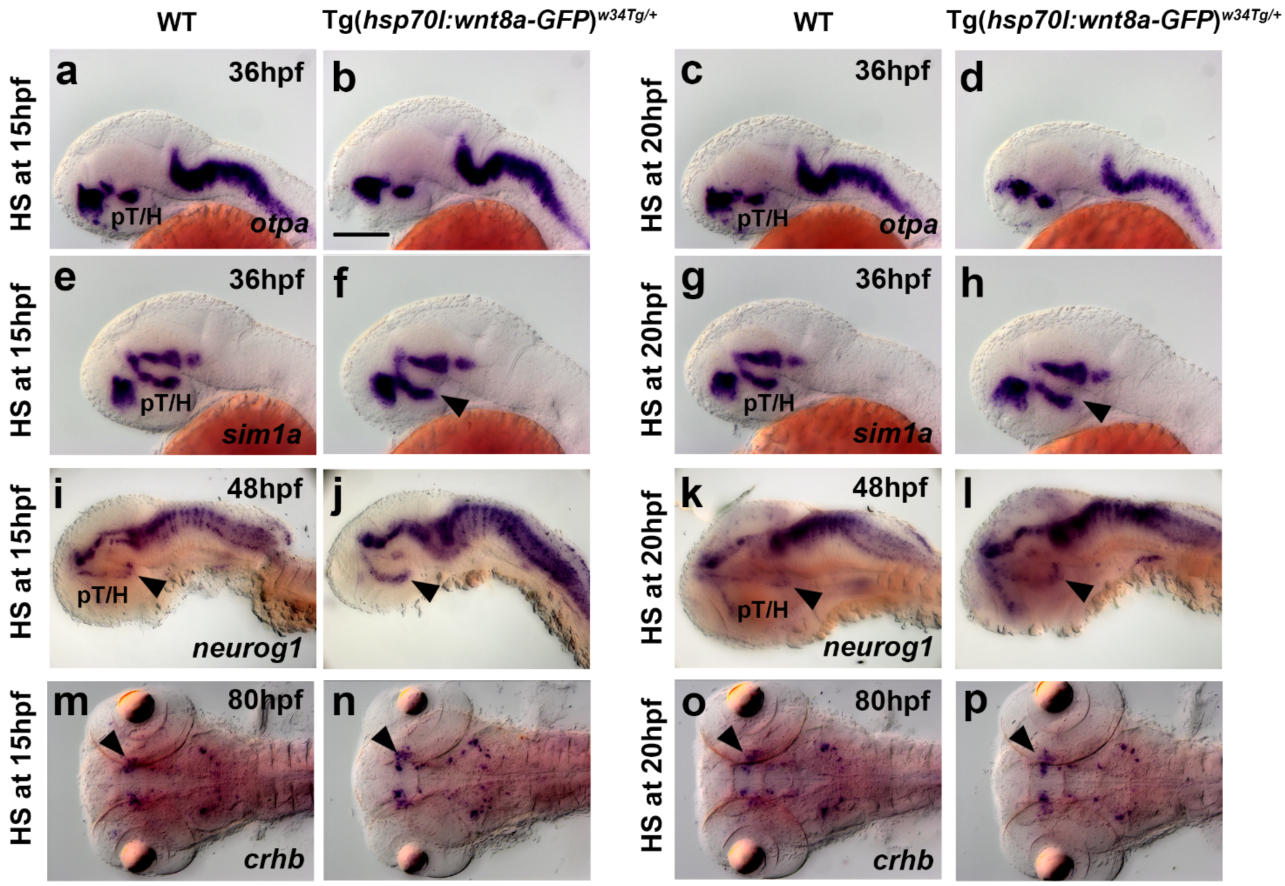

Supplementary Figure 7: Effects of heat-shock induced overexpression of Wnt8a on neurogenesis and neuron markers in the ventral diencephalon and hypothalamus.

(a-p) Expression analysis of *otpa* (a-d), *sim1a* (e-h), *neurog1* (i-l) and *crhb* (m-p) as detected by WISH in WT embryos (a, c, e, g, I, k,, m, o) and in heterozygous transgenic Tg(*hsp70l:wnt8a-GFP*)$^{w34Tg/+}$ siblings (b, d, f, h,j, l, n, p) after heat-shock treatments at stages indicated at left of each image pair, and subsequently fixed at stages indicated in image panels. Lateral views (a-l) and dorsal views (m-p) of larval heads, images show Z-Projections of image stacks. Abbreviations: H: hypothalamus; pT: posterior tuberculum; vDC: ventral diencephalon. Scale bar in (b) is 100 µm for all images.

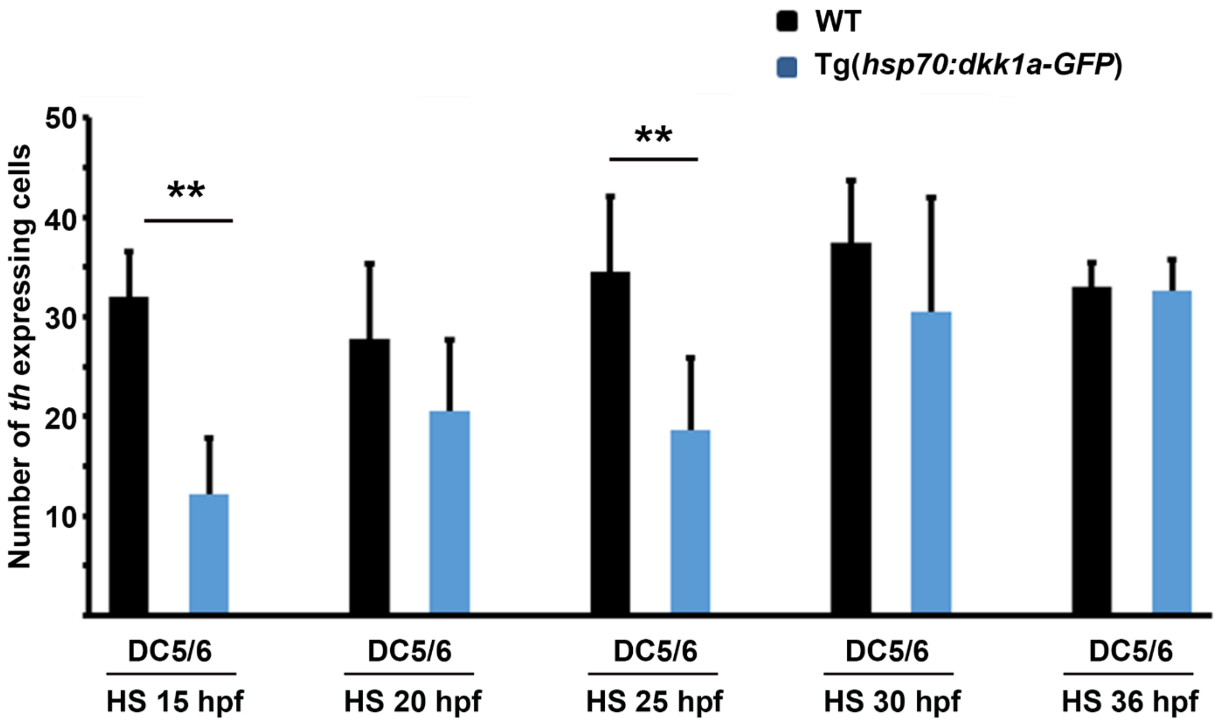

Supplementary Figure 8: Effects of heat-shock induced Dkk1b overexpression on *th*-expressing cell numbers.

Quantification of *th*-expressing cells in WT embryos compared to heterozygous transgenic Tg(*hsp70l:dkk1-GFP*)$^{32Tg/+}$ siblings heat shocked at either 15 hpf, 20 hpf, 25 hpf, 30 hpf or 36 hpf, and fixed at 82 hpf. Bar graphs show the mean cell count numbers of *th*-expressingcells for DC5/6 DA neuron groups. Error bars depict standard deviations of the mean (Mann-Whitney U test; p=0.0095 for heat-shoch treatments at 15 hpf and 25 hpf; WT sibling n=4 and transgenic larvae n=6 in each condition). Representative images of WISH for *th* in WT and heterozygous Tg(*hsp70l:dkk1-GFP*)$^{32Tg/+}$ siblings are shown in Figure 7c-l. Software: Microsoft Excel Version 16.54.

	**TH immunoreactive DC 5/6 cell numbers analyzed at 82 hpf**									
	**HS 15 hpf**		**HS 20 hpf**		**HS 25 hpf**		**HS 30 hpf**		**HS 36 hpf**	
	WT	GFP+	WT	GFP+	WT	GFP+	WT	GFP+	WT	GFP+
e01	31	8	34	29	27	7	34	12	35	30
e02	34	8	24	25	29	15	31	35	32	36
e03	31	11	25	18	41	17	40	22	30	31
e04	32	16	28	18	41	25	45	40	35	36
e05		18		20		22		32		34
e06		12		13		26		42		29

The table shows the cell counts for each of the 4 respectively 6 embryos e01 to e06 per experimental condition. The heat-shock induced reduction of *th*-expressing cells in DC5 and DC6 is less pronounced in embryos heat-shocked at 20 hpf, as compared to embryos heat-shocked at either 15 or 25 hpf, however we think this may reflect that some of the WT control embryos at HS 20hpf had relatively low *th*-expressing cell counts.

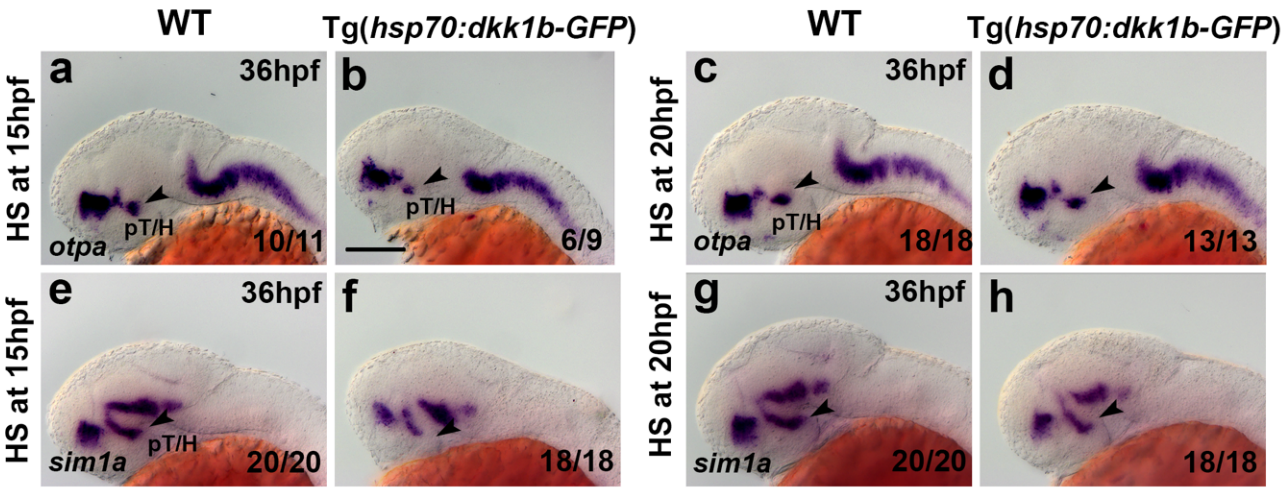

Supplementary Figure 9: Effects of heat-shock induced overexpression of Dkk1b on neurogenesis in the ventral diencephalon and hypothalamus.

(a-l) Expression analysis of *otpa* (a-d) and *sim1a* (e-h), as detected by WISH in WT embryos and in heterozygous transgenic Tg(*hsp70l:dkk1b-GFP*)$^{w32Tg/+}$ siblings after heat-shock treatment as indicated at left of each image pair, and subsequently fixed at 36 hpf. Lateral views of heads of larvae, images show Z-projections of image stacks. Abbreviations: H: hypothalamus; pT: posterior tuberculum. Scale bar in (b) is 100 µm for the whole panel. Numbers N/N indicate number of representative phenotypes as shown in image versus total embryos analyzed for this condition.

Supplementary Videos

Supplementary Video 1: Expression of *wnt8b* in relation to dopaminergic neurons in the ventral diencephalon and hypothalamus at 24 hpf: Expression of *wnt8b* detected by fluorescent *in situ* hybridization (green) in relation to TH-immunoreactive DA neurons detected by immunofluorescence (red) in an embryo at 24 hpf. Dorsal views of the ventral diencephalon/hypothalamus region. Confocal image stacks were recorded and video shows the complete Z- stack. Single optical sections of Z- stacks containing TH-immunoreactive cells are shown in Figure 1(g).

Supplementary Video 2: Expression of *wnt8b* in relation to dopaminergic neurons in the ventral diencephalon and hypothalamus at 48 hpf: Expression of *wnt8b* detected by fluorescent *in situ* hybridization (green) in relation to TH-immunoreactive DA neurons detected by immunofluorescence (red) in an embryo at 48 hpf. Dorsal views of the ventral diencephalon/hypothalamus region. Confocal image stacks were recorded and video shows the complete Z- stack. Single optical sections of Z- stacks containing TH-immunoreactive cells are shown in Figure 1 (h-i)

Supplementary Video 3 : Expression of *wnt8b* in relation to dopaminergic neurons in the ventral diencephalon and hypothalamus at 72 hpf: Expression of *wnt8b* detected by fluorescent *in situ* hybridization (green) in relation to TH-immunoreactive DA neurons detected by immunofluorescence (red) in an embryo at 72 hpf. Dorsal views of the ventral diencephalon/hypothalamus region. Confocal image stacks were recorded and video shows the complete Z- stack. Single optical sections of Z- stacks containing TH-immunoreactive cells are shown in Figure 1 (j-l).

Supplementary Video 4: Activity domains of Wnt/ß-catenin signaling in relation to TH-immunoreactive cells in the ventral diencephalon and hypothalamus at 26 hpf.
Wnt/ß-catenin-reporter Tg(*7xtcf-Xla.siam:GFP*) zebrafish embryos were stained by double immunofluorescence for TH-immunoreactive cells (red) and GFP-immunoreactive cells (green) at 26 hpf. Dorsal views of the ventral diencephalon/hypothalamus region. Confocal image stacks were recorded and video shows the complete Z- stack. Single optical sections of Z- stacks containing TH-immunoreactive are cells shown in Figure 2 (d).

Supplementary Video 5: Activity domains of Wnt/ß-catenin signaling in relation to TH-immunoreactive cells in the ventral diencephalon and hypothalamus at 48 hpf.
Wnt/ß-catenin-reporter Tg(*7xtcf-Xla.siam:GFP*) zebrafish embryos were stained by double immunofluorescence for TH-immunoreactive cells (red) and GFP-immunoreactive cells (green) at 48 hpf. Dorsal views of the ventral diencephalon/hypothalamus region. Confocal image stacks were recorded and video shows the complete Z- stack. Single optical sections of Z- stacks containing TH-immunoreactive cells are shown in Figure 2 (e-f).

Supplementary Video 6: Activity domains of Wnt/ß-catenin signaling in relation to TH-immunoreactive cells in the ventral diencephalon and hypothalamus at 72 hpf.
Wnt/ß-catenin-reporter Tg(*7xtcf-Xla.siam:GFP*) zebrafish embryos were stained by double immunofluorescence for TH-immunoreactive cells (red) and GFP-immunoreactive cells (green) at 72 hpf. Dorsal views of the ventral diencephalon/hypothalamus region. Confocal image stacks were recorded and video shows the complete Z- stack. Single optical sections of Z- stacks containing TH-immunoreactive cells are shown in Figure 2 (g-i).

Supplementary Video 7: Activity domains of Wnt/ß-catenin signaling in relation to TH-immunoreactive cells in the ventral diencephalon and hypothalamus at 96 hpf.
Wnt/ß-catenin-reporter Tg(*7xtcf-Xla.siam:GFP*) zebrafish embryos were stained by double immunofluorescence for TH-immunoreactive cells (red) and GFP-immunoreactive cells (green) at 96 hpf. Dorsal views of the ventral diencephalon/hypothalamus region. Confocal image stacks were recorded and video shows the complete Z- stack. Single optical sections of Z- stacks containing TH-immunoreactive cells are shown in Figure 2 (j-l).

Supplementary References

1 Krauss, S., Korzh, V., Fjose, A. & Johansen, T. Expression of four zebrafish wnt-related genes during embryogenesis. *Development* **116**, 249-259 (1992).

2 Duncan, R. N., Panahi, S., Piotrowski, T. & Dorsky, R. I. Identification of Wnt Genes Expressed in Neural Progenitor Zones during Zebrafish Brain Development. *PLoS One* **10**, e0145810, doi:10.1371/journal.pone.0145810 (2015).

3 Kelly, G. M., Greenstein, P., Erezyilmaz, D. F. & Moon, R. T. Zebrafish wnt8 and wnt8b share a common activity but are involved in distinct developmental pathways. *Development* **121**, 1787-1799 (1995).

4 Ungar, A. R. & Calvey, C. R. Zebrafish frizzled7b is expressed in prechordal mesoderm, brain and paraxial mesoderm. *Mech Dev* **118**, 165-169, doi:10.1016/s0925-4773(02)00221-6 (2002).

5 Kim, S. H. *et al.* Characterization of two frizzled8 homologues expressed in the embryonic shield and prechordal plate of zebrafish embryos. *Mech Dev* **78**, 193-201, doi:10.1016/s0925-4773(98)00137-3 (1998).

6 Momoi, A. *et al.* Analysis of Wnt8 for neural posteriorizing Frizzled 8c and Frizzled 9 as functional factor by identifying receptors for Wnt8. *Mech Develop* **120**, 477-489, doi:10.1016/S0925-4773(03)00003-0 (2003).

7 Pezeron, G. *et al.* Duplicate sfrp1 genes in zebrafish: sfrp1a is dynamically expressed in the developing central nervous system, gut and lateral line. *Gene Expression Patterns* **6**, 835-842, doi:10.1016/j.modgep.2006.02.002 (2006).

8 Tendeng, C. & Houart, C. Cloning and embryonic expression of five distinct sfrp genes in the zebrafish Danio rerio. *Gene Expr Patterns* **6**, 761-771, doi:10.1016/j.modgep.2006.01.006 (2006).

9 Hsieh, J. C. *et al.* A new secreted protein that binds to Wnt proteins and inhibits their activities. *Nature* **398**, 431-436, doi:Doi 10.1038/18899 (1999).

10 Hashimoto, H. *et al.* Zebrafish Dkk1 functions in forebrain specification and axial mesendoderm formation. *Dev Biol* **217**, 138-152, doi:10.1006/dbio.1999.9537 (2000).

11 Mahler, J., Filippi, A. & Driever, W. DeltaA/DeltaD regulate multiple and temporally distinct phases of notch signaling during dopaminergic neurogenesis in zebrafish. *J Neurosci* **30**, 16621-16635, doi:10.1523/JNEUROSCI.4769-10.2010 (2010).
